# The Immune Subtypes and Landscape of Gastric Cancer and to Predict Based on the Whole-Slide Images Using Deep Learning

**DOI:** 10.3389/fimmu.2021.685992

**Published:** 2021-06-28

**Authors:** Yan Chen, Zepang Sun, Wanlan Chen, Changyan Liu, Ruoyang Chai, Jingjing Ding, Wen Liu, Xianzhen Feng, Jun Zhou, Xiaoyi Shen, Shan Huang, Zhongqing Xu

**Affiliations:** ^1^ Department of General Practice, Tongren Hospital, Shanghai Jiao Tong University, School of Medicine, Shanghai, China; ^2^ Department of Endocrinology, Tongren Hospital, Shanghai Jiao Tong University, School of Medicine, Shanghai, China; ^3^ Department of General Surgery, Nanfang Hospital, Southern Medical University, Guangzhou, China; ^4^ Guangdong Provincial Key Laboratory of Precision Medicine for Gastrointestinal Tumor, Guangzhou, China; ^5^ Department of Cardiology, Tongren Hospital, Shanghai Jiao Tong University, School of Medicine, Shanghai, China

**Keywords:** tumor-infiltrating immune cells, immune subtypes, immunotherapy, deep learning, gastric cancer

## Abstract

**Background:**

Gastric cancer (GC) is a highly heterogeneous tumor with different responses to immunotherapy. Identifying immune subtypes and landscape of GC could improve immunotherapeutic strategies.

**Methods:**

Based on the abundance of tumor-infiltrating immune cells in GC patients from The Cancer Genome Atlas, we used unsupervised consensus clustering algorithm to identify robust clusters of patients, and assessed their reproducibility in an independent cohort from Gene Expression Omnibus. We further confirmed the feasibility of our immune subtypes in five independent pan-cancer cohorts. Finally, functional enrichment analyses were provided, and a deep learning model studying the pathological images was constructed to identify the immune subtypes.

**Results:**

We identified and validated three reproducible immune subtypes presented with diverse components of tumor-infiltrating immune cells, molecular features, and clinical characteristics. An immune-inflamed subtype 3, with better prognosis and the highest immune score, had the highest abundance of CD8+ T cells, CD4+ T–activated cells, follicular helper T cells, M1 macrophages, and NK cells among three subtypes. By contrast, an immune-excluded subtype 1, with the worst prognosis and the highest stromal score, demonstrated the highest infiltration of CD4+ T resting cells, regulatory T cells, B cells, and dendritic cells, while an immune-desert subtype 2, with an intermediate prognosis and the lowest immune score, demonstrated the highest infiltration of M2 macrophages and mast cells, and the lowest infiltration of M1 macrophages. Besides, higher proportion of EVB and MSI of TCGA molecular subtyping, over expression of CTLA4, PD1, PDL1, and TP53, and low expression of JAK1 were observed in immune subtype 3, which consisted with the results from Gene Set Enrichment Analysis. These subtypes may suggest different immunotherapy strategies. Finally, deep learning can predict the immune subtypes well.

**Conclusion:**

This study offers a conceptual frame to better understand the tumor immune microenvironment of GC. Future work is required to estimate its reference value for the design of immune-related studies and immunotherapy selection.

## Introduction

Gastric cancer (GC) is the fifth most common malignant tumor and third leading cause of cancer-related death worldwide (1). Despite major advancements in therapies, the 5-year overall survival (OS) rate for patients in advanced stage remains 20% (2). Even patients with locally advanced disease underwent radical resection and perioperative chemotherapy, the 5-year OS rate is still less than 40% (3–7). Thus, more effective systemic treatments are obviously urgent.

Immunotherapy is catching attention in multiple solid tumors recently, including gastric cancer. Specifically, immune checkpoint inhibitors, such as cytotoxic T-lymphocyte associated protein 4 (CTLA4) antibodies and programmed cell death protein 1 (PD1) antibodies, presented unprecedented clinical benefit in a variety of tumors (8–18). However, for patients with advanced gastric cancer, only a small subset (10–20%) responded to anti-CTLA4 (ipilimumab) and anti-PD1 (nivolumab, pembrolizumab) (8–12). A randomized controlled phase 3 trial ONO-4538-12/ATTRACTION-2 indicates an improvement of objective response rate (ORR) of 11% for patients with advanced gastric cancer receiving nivolumab versus placebo (10). Also, the ORR remains similar for other clinical trials, including the phase 1b KEYNOTE-012 (ORR 22%) and phase II KEYNOTE-059 (ORR 12%) trials (9, 11). Therefore, researches to identify mechanisms of response and resistance to immune checkpoint inhibition and to screen underlying patients who may benefit are required. However, our understanding of the role of tumor microenvironment (TME) in immune response remains incomplete because of its complexity.

The tumor microenvironment is a complex system composed of extracellular matrix, cytokines, chemokines, and non-tumor cells (19). As an important component of non-tumor cells in TME, tumor infiltrating immune cells (TIICs) is associated with the promotion or inhibition of tumor growth (20–22). In particular, the presence of tumor-associated CD8+ T cells, CD4+ T cells, T follicular helper cells (Tfhs), and natural killer (NK) cells in TME, suggesting activated immune response, is associated with good prognosis, while regulatory T cells (Tregs), B cells, macrophages, mast cells and plasma cells inhibiting immune response indicate poor prognosis (22–31). Conventional detection techniques for TIICs, such as flow cytometry and immunohistochemistry, are generally confined to evaluate limited types of immune cells, due to inability to measure numbers of markers simultaneously (29, 32). However, the interactions among tumor-infiltrating immune cells are extremely complicated. Thus, a systematic assessment of all immune cells in the TME offers better clinical value.

Immune subtypes have presented with meaningful clinical value in multiple tumors, including melanoma, esophageal cancer, lung cancer, and breast cancer (33, 34). Although the relationship between tumor infiltrating immune cells and gastric cancer has been described, the overall function of TME is ignored (35). Therefore, our understanding of the immune subtypes based on TIICs in gastric cancer is far from complete. From this perspective, our study is of great significance.

Deep learning performs excellently as a powerful technique for reading pathological images (36, 37). The emergence of pathological scanning copy for the whole slide images (WSIs) provides a platform for deep learning (34, 37). It is generally acknowledged that the histopathology images contain valuable information of TME (38). Therefore, deep learning could extract high dimensional data from standard medical images for clinical applications, such as distinguishing immune subtypes. Besides, convincing performance for deep learning has been observed in prediction of microsatellite instability status, immune cell types and prognosis in a variety of tumors (39–42), which provides reference for our study.

In the present study, we identified three robust immune subtypes of gastric cancer based on unsupervised consensus clustering of TIICs, and their reproducibility was further validated in an independent cohort. We observed that each of the three immune subtypes presented distinct immune cells proportion, molecular features, and clinical characteristics, which could provide reference for the design of immune-related studies and the choice of immunotherapy. Moreover, we verified the feasibility and prognostic value of this classification system in five pan-cancer data sets, including breast cancer, esophageal cancer, colorectal cancer, liver cancer, and pancreatic cancer. Finally, we developed and validated a deep learning model based on pathological images to predict the immune subtypes for easy-use in clinical practice.

## Materials and Methods

### Patients and Data Sets

The discovery cohort to identify the immune subtypes consisted of 375 patients with gastric cancer obtained from The Cancer Genome Atlas (TCGA) database (https://cancergenome.nih.gov). Another cohort including 433 patients with gastric cancer in GSE84437 downloaded from the Gene Expression Omnibus (GEO) database was used to validate the immune subtypes (https://www.ncbi.nlm.nih.gov/geo/). Besides, five independent cohorts (total n = 2230), including breast cancer (n=1108), esophageal cancer (n=185), colorectal cancer (n=383), liver cancer (n=375), and pancreatic cancer (n=179), acquired from UCSC Xena (https://xenabrowser.net/) were applied to further elucidate feasibility of the immune subtypes. For details about study design and data preprocessing, please refer to supplementary methods and **Figure S1**.

### Data Processing and Quantification of Immune Cells

Based on the gene expression profiles, the CIBERSORT algorithm was employed to quantify the proportions of 22 Tumor-infiltrating immune cells using the LM22 signature and 1,000 permutations (43). Cases with P<0.05 in CIBERSORT, which indicated that the deconvolution results were accurate, would be retained for further analysis. In this study, a total of 194 GC samples from discovery cohort and 299 GC samples from validation cohort were screened out (**Figure S2**). Finally, we obtained 22 types of immune cells, including B cells (naive B cells and memory B cells), CD8+ T cells, naive CD4+ T cells, resting memory CD4+ T cells, activated memory CD4+ T cells, T follicular helper cells (Tfh), regulatory T cells (Tregs), natural killer cell (resting NK cells, activated NK cells), macrophages (M0, M1 and M2), dendritic cells (resting DC and activated DC), mast cells (resting mast cells and activated mast cells), plasma cells, gamma delta T cells, monocytes, neutrophils, and eosinophils (**Figure S3**).

### Discovery and Validation of the Immune Subtypes

To dissect inter-tumor heterogeneity defined by TIICs, we applied unsupervised consensus clustering to define the robust subgroup of patients, i.e., immune subtypes. Specifically, the K-Means clustering algorithm with the Euclidean distance metric and performed 10,000 bootstraps, with 80% resampling of the immune cells. The consensus clustering algorithm was implemented with the ConsensusClusterPlus package (44). The number of clusters was determined by the optimal consensus matrix and explicit cluster allocation across permuted runs. Besides, in order to evaluate the reproducibility of the clusters, the same clustering procedure was performed independently in the validation cohort. We then calculated the in-group proportion (IGP) index with “clusterRepro” R package to quantitatively measure the similarity of clusters produced from the two data sets (45).

### Assessing the Clinical, Molecular, Cellular Characteristics Associated With the Immune Subtypes

We first evaluated the association of immune-related cellular features with immune subtypes using Kruskal-Wallis statistic. TIICs (naive CD4+ T cells, gamma delta T cells, monocytes, neutrophils, and eosinophils) with zero value in more than 40% of all samples were excluded from the analysis. Next, we described the distribution of demographic, clinicopathological characteristics, and molecular feature of the immune subtypes, including age, sex, Lauren’s classification, pathological differentiation status, tumor location, stage, TCGA molecular subtyping, and stromal-immune score based on ESTIMATE algorithm (46, 47). Finally, log-rank test and multivariable Cox regression were used to measure the prognostic value of the immune subtypes with OS as the endpoint. For details about identification of TCGA molecular subtyping, please refer to **Supplementary Methods**.

### Validation Using Pan-Cancer Data Set

Tumor-infiltrating immune cells data were extracted based on the CIBERSORT method described above from the pan-cancer data sets (breast cancer, esophageal cancer, colorectal cancer, liver cancer, and pancreatic cancer). However, for those cohorts with too few samples, we chose P<0.1 as the cutoff point. Then, the consensus clustering algorithm and Kaplan-Meier analysis were performed to illustrate the feasibility of our immune subtypes.

### Functional Enrichment Analyses for Immune Subtypes

Differentially expressed genes (DEGs) were identified between any two immune subtypes (IS1 *vs* IS2, IS1 *vs* IS3, IS2 *vs* IS3) using an R package “limma”. An absolute value of log2 (fold change) >1 combined with the false discovery rate (FDR) adjusted p-value <0.05 was selected as the threshold for DEG identification. The intersection of the DEGs in TCGA-GC cohort and GSE84437 cohort was applied to Gene Ontology (GO), Kyoto Encyclopedia of Genes and Genomes (KEGG) and Gene Set Enrichment Analysis (GSEA). For the enrichment analysis, we focused on the immune related gene sets and cancer hallmark gene sets. Besides, several classic immune checkpoints (PD1, PDL1, and CTLA4) and cancer related genes (TP53, JAK1) were evaluated among the immune subtypes.

### Deep Learning to Identify Immune Subtypes

Deep learning can identify the macroscopic contents of pathological images, including tumor cells and TIICs nuclear size, nuclear location, nuclear morphology, etc. It can even identify high-dimensional data, such as color matrix, histogram matrix, and high-order matrix, which cannot be distinguished by naked eye. Thus, we trained a convolutional neural network with deep residual learning (based on ResNet-18) model to detect the immune subtype by transfer learning using patches segmented from the whole slide images (WSIs). First, high-quality WSIs without obvious interfering factors, including bleeding, creases, necrosis, and blurred areas, were screened and divided into training, validation and test sets at a 5:3:2 ratio for further processing. Next, tumor regions of interest (ROIs) on WSIs were manually delineated by expert pathologists. All WSIs were digitalized at 20× objective lens. Then, ROIs were subsequently separated into 512 pixels × 512 pixels patches. Finally, after preprocessed with random cutting, random horizontal flipping, and random affine transformation, center cropping (224 pixels × 224 pixels), and normalization, patches were put into the deep learning model based on ResNet-18. For details about data preprocessing, please refer to **Supplementary Methods**.

### Statistical Analysis

All the statistical significance values were set as two-tail and P < 0.05 was considered statistically significant. Statistical analyses were performed using SPSS statistical software (version 22.0), GraphPad Prism software (version 7.00), Perl 5 software (version 5.28.1) and R software (version 3.5.3). Deep learning was implemented with torch library in Python software (version 3.6.7).

## Results

### Immune Subtypes Discovery and Validation

**Figure 1:**
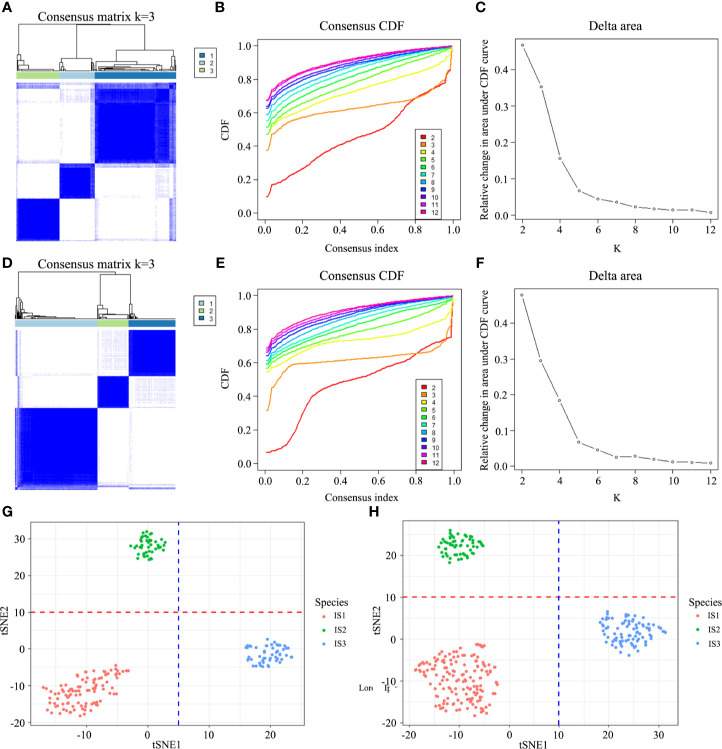
Discovery and validation of the immune subtypes in TCGA **(A)** and GEO **(D)**. Patient samples are both in rows and columns, and consensus values range from 0 (never clustered together) to 1 (always clustered together). The optimal cluster number (K = 3) is determined by the area under the cumulative distribution function (CDF) curve in the discovery **(B, C)** and validation cohort **(E, F)**, which corresponds to the largest number of clusters that induced the smallest incremental change in the area under the CDF curves. The tSNE well represents the discrete distribution of three clusters **(G, H)**.

By performing the unsupervised consensus clustering on the 194 GC cases from TCGA based on the 22 TIICs, the optimal number of clusters was found to be three with maximal consensus within clusters and minimal ambiguity among clusters (**Figures 1A–C**). Based on this, we identified three robust immune subtypes—immune subtype 1 (IS1), immune subtype 2 (IS2) and immune subtype 3 (IS3). To evaluate the reproducibility of the immune subtypes, we performed the same algorithm in the 299 GC cases from GSE84437. Interestingly, we found that the optimal number of clusters was three, too (**Figures 1D–F**). The tSNE analysis well represented the discrete distribution of three clusters and the consistency of the discovery and validation cohorts (**Figures 1G, H** **)**. Furthermore, we calculated the in-group proportion (IGP) statistic to quantify the similarity of the immune subtypes between the discovery and validation cohort. And immune subtypes showed good consistency between the two cohorts, with corresponding IGP value at 79%, 81%, and 86% in IS1, IS2, and IS3, respectively.

### Profound Differences in Immune Infiltration Among Immune Subtypes

**Figure 2:**
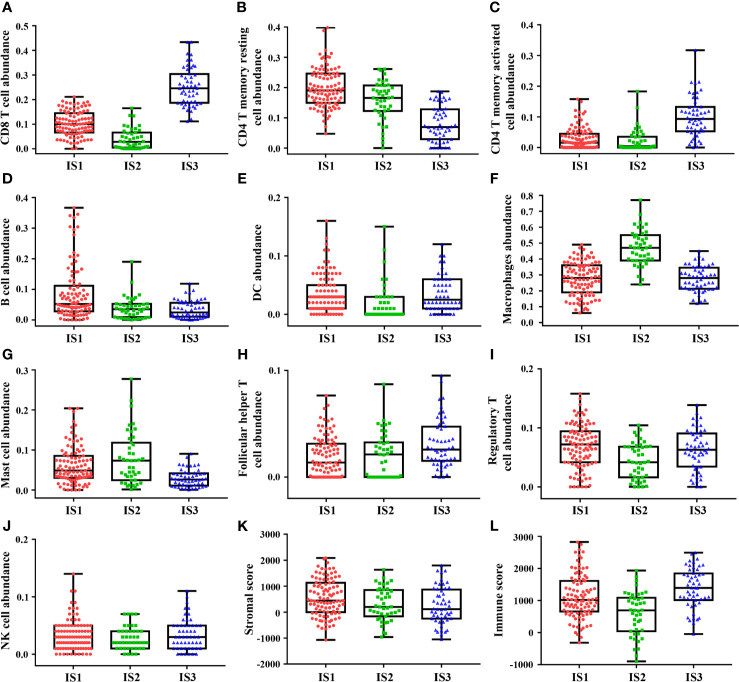
The discovery cohort shows heterogeneity of immune infiltration among immune subtypes. Highest abundance of CD8+ T cells, CD4+ T memory activated cells, follicular helper T cells, and NK cells was observed in IS3 **(A, C, H, J)**, while highest abundance of CD4+ T memory resting cells, B cells, macrophages cells, and mast cells was observed in IS3 **(B, D, F, G)**. DC cells and regulatory T cells abundance showed one highest and one lowest in IS1 and IS2 **(E, I)**. Besides, IS3 exhibited the lowest stromal score and highest immune score **(K, L)**. The plot of patient immune cells abundance shows the median, 25th and 75th percentile values (horizontal bar, bottom, and top bounds of the box), and the highest and lowest values (top and bottom whiskers, respectively).

Each of the three immune subtypes represented distinct immune cells expression patterns in the discovery cohort, which was found to be highly consistent with the validation cohort, surprisingly. Highest CD8+ T cells and M1 macrophages abundance was confirmed in IS3 (**Figures 2A** and **S5B**). IS3 was also characterized by the highest abundance of activated CD4+ T memory cells (**Figure 2C**). However, the least abundance of resting CD4+ T memory cells and M2 macrophages presented in IS3 (**Figures 2B** and **S5C**). Moreover, IS3 was also associated with highest abundance of Tfh and NK cells (**Figures 2H, J** **)** and lowest abundance of B cells and mast cells **(** **Figures 2D, G** **)**. Besides, the expression of DC and Tregs of IS3 was in the middle among three immune subtypes **(** **Figures 2E, I** **)**, whereas the expression of plasma cells in IS3 was high in the discovery cohort and low in the validation cohort (**Figure S4I, K**). In comparison, IS1 exhibited the highest density of CD4+ T memory resting cells, B cells, DC cells, and Tregs **(** **Figures 2B, D, E, I**), and the lowest density of macrophages and Tfh (**Figures 2F, H** **)**. The expression of CD8+ T cells of IS1 was in the middle among three immune subtypes **(** **Figure 2A** **)**. Furthermore, compared with IS1 and IS3, the highest abundance of macrophages in IS2 was confirmed (**Figure 2F**), accompanied with the lowest abundance of CD8+ T cells, DC and Tregs (**Figures 2A, E, I**). Besides, the highest abundance of M0 and M2 macrophages was observed in IS2 **(** **Figures S5A, C, D, F** **),** while the lowest abundance of M1 macrophages was observed in IS2 compared with IS1 and IS3 (**Figure S5**). And the expression of CD4+ T memory resting cells, B cells, Tfh of IS2 was in the middle among three immune subtypes **(** **Figures 2B, D, H** **)**. The expression of activated CD4+ T memory cells, mast cells, NK cells was inconsistent in IS1 or IS2 between the discovery cohort and validation cohort **(** **Figures 2C**, **S4C**, **2G**, **S4G**, **2J** and **S4J** **)**. Additionally, comparison of TIICs between any two immune subtypes (IS1 *vs* IS2, IS1 *vs* IS3, IS2 *vs* IS3) was provided in supplementary results. Lastly, we identified that IS3 exhibited the lowest stromal score and highest immune score (**Figures 2K, L**), while IS1 exhibited the highest stromal score (**Figures 2K**) and IS2 exhibited the lowest immune score (**Figures 2L**). The results of these findings were consistent with the validation cohort (**Figures S4** and **S5** **)**. Specific values of TIICs abundance and their P values are presented in **Table 1**. These results suggested that IS3 had the strongest immune activity accompanied with a weaker immune-suppression (immune-inflamed phenotype), while IS1 had a moderate immune response accompanied by a stronger immune-suppression (immune excluded phenotype), while IS3 was characterized by immune deficiency (immune-desert phenotype).

**Table 1:** The proportion of tumor-infiltrating immune cells in patients with gastric cancer from TCGA and GEO.

Variables	TCGA				GEO			
	IS1 (n=99)	IS2 (n=43)	IS3 (n=52)	P value	IS1 (n=153)	IS2 (n=59)	IS3 (n=87)	P value
	median (IQR)	median (IQR)	median (IQR)		median (IQR)	median (IQR)	median (IQR)	
**B cell**	0.053 (0.029-0.117)	0.035 (0.010-0.053)	0.025 (0.011-0.056)	<0.001	0.055 (0.029-0.092)	0.042 (0.017-0.067)	0.024 (0.011-0.053)	<0.001
**Plasma cells**	0.039 (0.009-0.066)	0.018 (0.00-0.018)	0.028 (0.015-0.068)	0.089	0.015 (0.00-0.073)	0.002 (0.00-0.035)	0.024 (0.00-0.063)	0.0348
**CD8+ T cell**	0.100 (0.066-0.145)	0.028 (0.005-0.028)	0.246 (0.187-0.304)	<0.001	0.064 (0.025-0.117)	0.030 (0.008-0.061)	0.159 (0.111-0.222)	<0.001
**CD4+ T memory resting cells**	0.190 (0.149-0.246)	0.166 (0.122-0.207)	0.069 (0.029-0.128)	<0.001	0.208 (0.168-0.285)	0.130 (0.086-0.189)	0.050 (0.003-0.085)	<0.001
**CD4+ T memory activated cells**	0.016 (0.00-0.045)	0.004 (0.00-0.035)	0.093 (0.053-0.132)	<0.001	0.028 (0.003-0.063)	0.045 (0.00-0.098)	0.173 (0.121-0.224)	<0.001
**T follicular helper cells**	0.013 (0.00-0.031)	0.021 (0.00-0.033)	0.026 (0.015-0.047)	0.002	0.024 (0.00-0.046)	0.023 (0.001-0.051)	0.041 (0.018-0.070)	<0.001
**T regulatory cells**	0.072 (0.042-0.094)	0.042 (0.016-0.068)	0.063 (0.034-0.091)	0.001	0.002 (0.00-0.016)	0.006 (0.00-0.028)	0.00 (0.00-0.008)	<0.001
**NK cells**	0.029 (0.014-0.047)	0.021 (0.011-0.022)	0.030 (0.012-0.051)	0.515	0.026 (0.014-0.048)	0.037 (0.028-0.051)	0.046 (0.028-0.070)	<0.001
**Macrophages**	0.279 (0.189-0.361)	0.468 (0.393-0.551)	0.280 (0.214-0.345)	<0.001	0.223 (0.160-0.291)	0.474 (0.394-0.527)	0.243 (0.205-0.305)	<0.001
**DC cell**	0.028 (0.011-0.053)	0.004 (0.00-0.027)	0.026 (0.014-0.061)	<0.001	0.022 (0.012-0.035)	0.011 (0.00-0.029)	0.020 (0.011-0.045)	0.004
**Mast cell**	0.049 (0.031-0.086)	0.074 (0.025-0.118)	0.026 (0.011-0.041)	<0.001	0.159 (0.099-0.214)	0.089 (0.061-0.121)	0.080 (0.048-0.126)	<0.001

IS1, immune subtype 1; IS2, immune subtype 2; IS3, immune subtype 3.

### Clinical Characteristics, Molecular Features, and Prognoses of the Immune Subtypes

**Figure 3:**
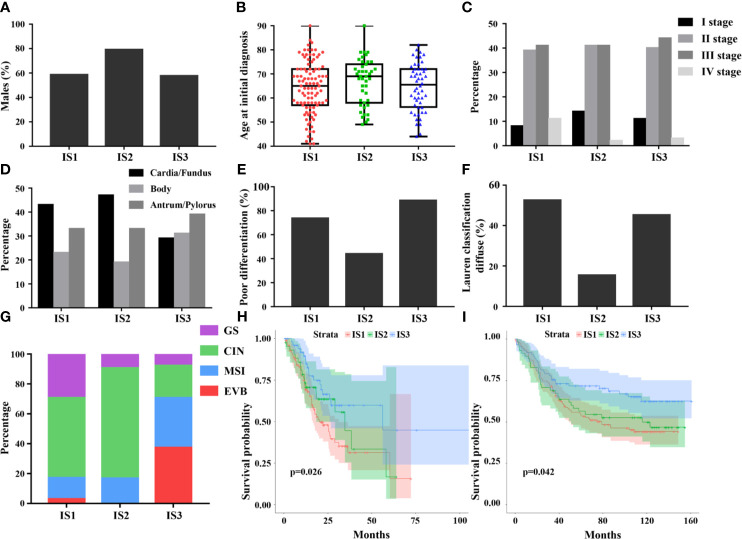
Differences in clinical and histological characteristics among immune subtypes, including age, sex, stage, tumor location, pathological differentiation, and Lauren classification **(A–F)**. The plot of patient age at initial diagnosis shows the median, 25th and 75th percentile values (horizontal bar, bottom, and top bounds of the box), and the highest and lowest values (top and bottom whiskers, respectively). The distribution of TCGA molecular subtyping among immune subtypes **(G)**. The prognostic value of the immune subtypes in TCGA **(H)** and GEO **(I)**, indicating best prognosis of IS3.

The TCGA cohort containing GC patients with available clinicopathologic information and molecular features, stratified by immune subtypes, was analyzed and listed in **Table 2**. Compared to IS1 and IS3, the median age of IS2 is slightly higher (**Figure 3B**). In addition, IS2 was associated with highest proportion of men and intestinal type tumor (**Figures 3A, F** **)**. Furthermore, IS3 was associated with a lower incidence of cardia/fundus cancer, while presented with worse pathological differentiation (**Figures 3D, E** **)**. Besides, there was no significant difference in the proportion of TNM stages among the three immune subtypes (**Figure 3C**). In terms of TCGA molecular subtyping, IS3 revealed more EVB and MSI, and less CIN and GS than that in 1 and 2 (**Figure 3G**). The clinicopathological information available in the validation cohort is listed in **Table S1** and **Figure S6**. Lastly, we observed that the immune subtypes revealed significantly prognostic impact in TCGA-GC and GEO cohort (**Figures 3H, I** **)**. Overall, the immune-hot subtype IS3 was associated with the best prognosis for OS among all subtypes. By contrast, the immune-cold subtype IS1 and IS2 was associated with poor outcomes. This survival difference was confirmed after excluding confounding factors of age, gender, tumor location, Lauren’s classification, pathological differentiation and stage and was showed in **Tables 3** and **S2**.

**Table 2:** Clinicopathological characteristics of patients with gastric cancer in TCGA.

Variables	TCGA							
	IS1 (n=99)		IS2 (n=43)			IS3 (n=52)		
	N	%	N		%	N		%
**Age (median, IQR, Y)**	65 (57-72)		69 (58-75)			65 (56-75)		
**Gender**								
Male	58	58.6	34		79.1	30		57.7
Female	41	41.4	9		20.9	22		42.3
**Lauren’s type**								
Intestinal	29	47.5	22		84.6	17		54.8
Diffuse	32	52.5	4		15.4	14		45.2
Unknown	38	NA	17		NA	21		NA
**Differentiation**								
Well	26	26.3	24		55.8	6		11.5
Poor	73	73.7	19		44.2	46		88.5
**Location**								
Cardia/Fundus	41	43.2	20		47.6	15		29.4
Body	22	23.2	8		19.1	16		31.4
Antrum/Pylorus	32	33.6	14		33.3	20		39.2
Unknown	4	NA	1		NA	1		NA
**Stage**								
I	8	8.1	6		14.0	6		11.5
II	39	39.4	18		41.9	21		40.4
III	41	41.4	18		41.9	23		44.2
IV	11	11.1	1		2.3	2		3.8
**Stromal score (median, IQR)**	445.4 (13.8-1104.2)		202.7 (-161-853.8)			113.9 (-248-853.2)		
**Immune score (median, IQR)**	1017.9 (659.1-1611.7)		694.7 (45.9-1086.7)			1394.3 (1016.3-1848.1)		
**Molecular characterization**								
EVB	3	3.0	0		0	19		37.3
MSI	14	14.1	7		16.7	17		33.3
CIN	53	53.5	31		73.8	11		21.6
GS	29	29.3	4		9.5	4		7.8
Unknown	0	NA	1		NA	1		NA

IS1, immune subtype 1; IS2, immune subtype 2; IS3, immune subtype 3; NA represents the meaningless values.

**Table 3:** Univariable and multivariable analyses for overall survival in patients with gastric cancer.

Variables	Univariable analysis (N=194)		Multivariable analysis (N=194)	
	OR (95%CI)	P	OR (95%CI)	P
**Age** (years)	1.018 (0.999-1.038)	0.061	NA	NA
**Gender** (female *vs*. male)	1.549 (0.984-2.437)	0.059	NA	NA
**Lauren type** (intestinal *vs* diffuse)	1.100 (0.859-1.409)	0.453	NA	NA
**Differentiation** (well *vs*. poor)	1.075 (0.671-1.723)	0.764	NA	NA
**Location** (cardia/fundus *vs*. body *vs*. antrum/pylorus)	0.865 (0.685-1.093)	0.224	NA	NA
**Stage**	1.399 (1.055-1.854)	0.020	1.371 (1.037-1.810)	0.026
**Stromal score**	1.020 (1.001-1.006)	0.086	NA	NA
**Immune score**	1.032 (1.000-1.144)	0.800	NA	NA
**Immune subtype**				
IS1	1	NA	1	NA
IS2	0.729 (0.425-2.148)	0.249	0.747 (0.435-1.282)	0.289
IS3	0.480 (0.277-0.834)	0.009	0.491 (0.282-0.853)	0.012

### Validation Using Pan-Cancer Data Set

The consensus clustering algorithm was conducted using the 22 TIICs based on patients in the pan-cancer data set (breast cancer, esophageal cancer, colorectal cancer, liver cancer, and pancreatic cancer). We observed that the optimal number of clusters was four in liver cancer, two in colorectal cancer, four in breast cancer, two in esophageal cancer, and two in pancreatic cancer. And survival difference was found in liver cancer, breast cancer, and pancreatic cancer. However, significant statistical differences were found only in liver cancer and pancreatic cancer. The total results were visualized in **Figure S8**.

### Functional Enrichment Analyses

**Figure 4:**
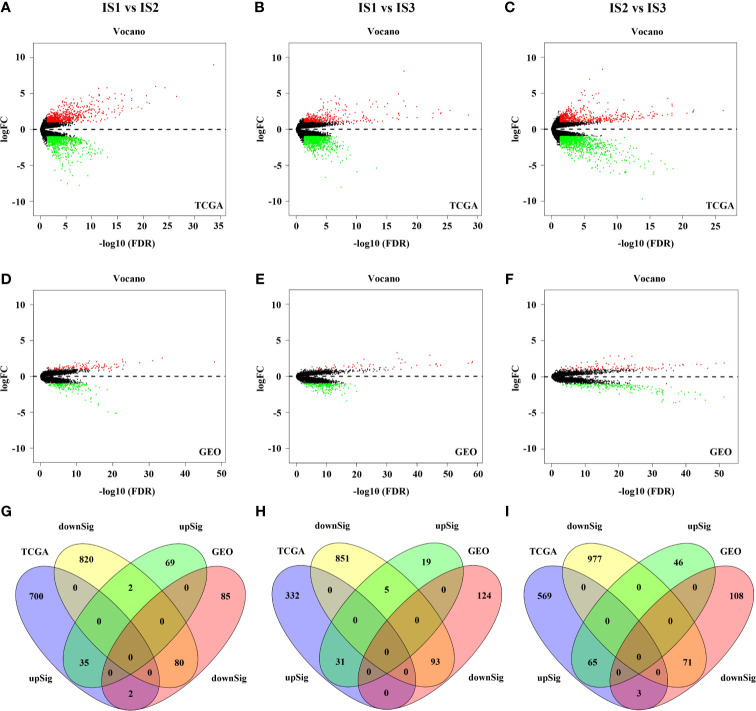
The results of differential expression analysis in the TCGA and GEO cohort **(A–F)**. 115 DEGs was found in IS1 *vs* IS2 **(G)**. 124 DEGs was found in IS1 *vs* IS3 **(H)**. 136 DEGs was found in IS2 *vs* IS3 **(I)**.

To investigate the underlying functional differences among immune subtypes, we conducted GO and GSEA analyses on the differentially expressed genes. Through the abovementioned analysis, GC cases in TCGA and GEO database were divided into three immune subtypes—IS1, IS2, and IS3. Thus, the functional enrichment analyses were performed between any two immune subtypes (IS1 *vs* IS2, IS1 *vs* IS3, IS2 *vs* IS3). First, 1639 DEGs, including 737 up-regulated expression (UE) and 902 down-regulated expression (DE), were filtered out from TCGA cohort and 208 DEGs (106 UE and 167 DE) were filtered out from GEO cohort in IS1 *vs* IS2 (**Figures 4A, D**, and **S7**). Next, a total of 115 DEGs (35 UE and 80 DE) were observed in the intersection between them (**Table S4** and **Figure 4G**). In IS1 *vs* IS3, 1312 DEGs (363 UE and 949 DE) were filtered out from TCGA and 272 DEGs (55 UE and 217 DE) were filtered out from GEO **(** **Figures 4B, E**, and **S7** **)**. Next, a total of 124 DEGs (31 UE and 93 DE) were observed in the intersection between them (**Table S5** and **Figure 4H**). In IS2 *vs* IS3, 1685 DEGs (637 UE and 1048 DE) were filtered out from TCGA and 293 DEGs (111 UE and 182 DE) were filtered out from GEO **(** **Figures 4C, F**, and **S7** **)**. Next, a total of 136 DEGs (65 UE and 71 DE) were observed in the intersection between them (**Table S6** and **Figure 4I**). Furthermore, GO, KEGG, and GSEA analyses were performed based on the DEGs separately (**Figures 4D, E** **)**.

**Figure 5:**
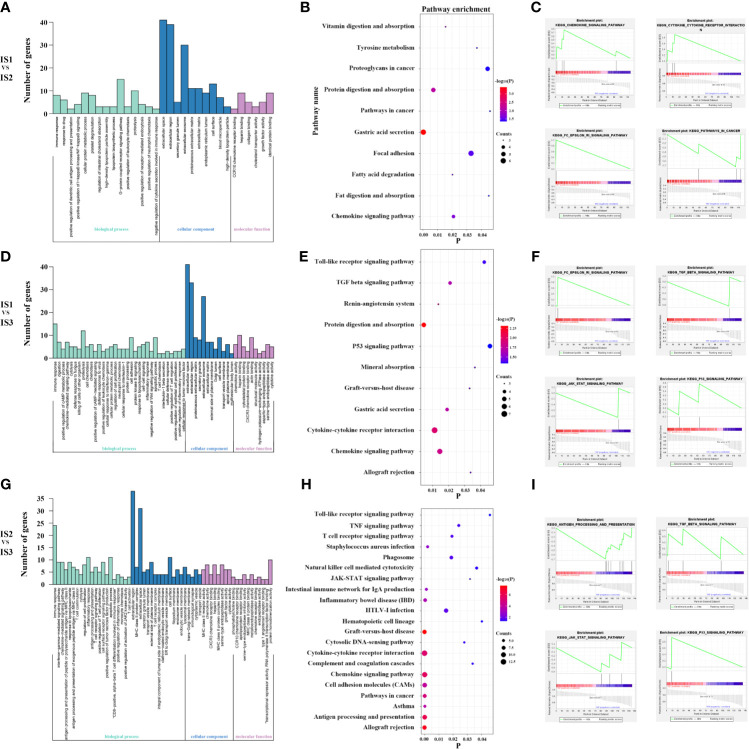
Lots of cytokine secretion and immune regulation pathways were found in the GO, KEGG and GSEA analysis for IS1 *vs* IS2 **(A–C)**. Lots of classical tumor and immune-related pathways were found in the GO, KEGG and GSEA analysis for IS1 *vs* IS3 **(D–F)**. Lots of classical tumor and immune-related pathways were found in the GO, KEGG, and GSEA analyses for IS2 *vs* IS3 **(G–I)**. These suggest more active immune respond and antitumor reaction in IS3.

From the above, we found significant difference of chemokine pathway between IS1 and IS2. IS presented with more active chemokine respondence and interactions (**Figures 5A–C**). Additionally, compared to IS3, TGF-β signaling was significantly enriched in IS1 and IS2, which suggested immunosuppression (**Figures 5F, I** **)**. Also, IS3 was associated with significantly upregulated T cell receptor signaling, antigen processing, and presentation signaling, suggesting that active inflammation and immune infiltration (**Figures 5D, E, G, H**). In addition, the classic tumor suppressor signaling P53 was observed to enrich in IS3 and the typical carcinogenic signaling JAK-STAT enriched in IS1-2 (**Figures 5F, I** **)**. The results consisted with the profound differences in immune infiltration among immune subtypes. And these may explain why IS3 has a better prognosis than IS1-2. Base on the above, we studied the relationship among several immune checkpoints (PD1, PDL1, and CTLA4), cancer related genes (TP53, JAK1) and the immune subtypes (**Table S3**). Interestingly, we found that compared with that in IS1-2, expression of PD1, PDL1, CTLA4, and TP53 was higher in IS3 and expression of JAK1 was lower, which was consistent with the functional enrichment analyses (**Figure S9**).

### Deep Learning Can Identify Immune Subtypes

**Figure 6:**
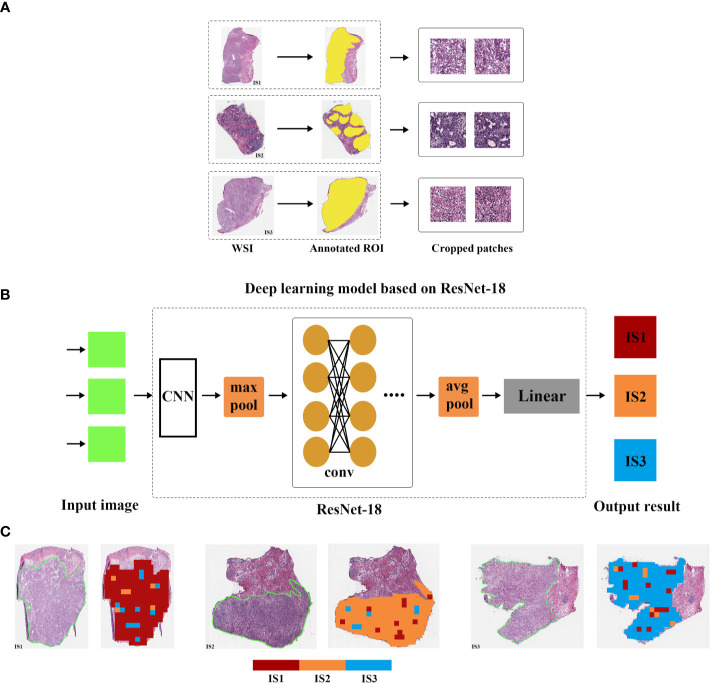
Overview of the deep learning model. The whole slide image (WSI) of each patient was obtained and annotated with regions of carcinoma (ROI) **(A)**. Then, tumor of ROI was segmented into patches, and the immune subtypes likelihood of each patch was predicted by deep learning model based on ResNet-18 **(B)**. Finally, multiple patch-level IS likelihoods were integrated into a WSI-level IS prediction **(C)**.

After removing low quality pathological images, 169 samples with WSIs were divided into training (84 cases), validation (51 cases), and test cohorts (34 cases), and then tumor ROI was separated into 512 × 512 patches. Finally, the training cohort contained 12,986 normalized tiles marked as IS1, 3,399 normalized tiles marked as IS2 and 6,323 normalized tiles marked as IS3. The validation cohort contained 11,070 normalized tiles marked as IS1, 3,003 normalized tiles marked as IS2, 5114 normalized tiles marked as IS3. And the test cohort contained 5344 normalized tiles marked as IS1, 1,790 normalized tiles marked as IS2, and 2,508 normalized tiles marked as IS3. Next, we developed a ResNet-18 deep learning model to predict the immune subtypes based on training and validation, and measured the performance in the test cohort. The model first predicted the probability of immune subtypes for each patch. We found that the accuracy of IS prediction for each patch in the training, validation, and test cohort was 80.23%, 74.45%, and 68.89%, respectively. Then GC cases would be designated as one of the three subtypes (IS1 or IS2 or IS3) according to the accumulated number of patches in tumor ROI (**Figure 6**). We observed that the accuracy of IS prediction ResNet-18 model for GC cases was about 85.71%, 80.39%, 76.47% in the training, validation, and test cohorts, separately (**Figures S10A, C**). Additionally, we observed that the accuracy of IS3 prediction would increase to about 90% when IS1 and the two were combine as IS1-2 (**Figures S10D–F**). More details refer to supplementary results.

## Discussions

Immunotherapy is increasingly being recognized for its potential therapeutic effect on a variety of tumors. However, only a subset of patients has response or survival benefit to immunotherapy. This may be caused by our incomplete understanding of the tumor immune microenvironment. Thus, to better understand the tumor immune microenvironment and further to filter out patients suitable for immunotherapy are particularly important. Here, we present the identification and validation of three reproducible immune subtypes of GC in a retrospective study with multiple cohorts. We observe that each of the immune subtypes presented with distinct composition of tumor infiltrating immune cells, and hence demonstrated widely different modes in gene expression profiles, functional orientation, molecular feature and clinical characteristics. Moreover, validation of a pan-cancer cohort can reinforce the credibility of our results. Lastly, a deep learning model with good performance to predict the status of immune subtypes in gastric cancer based on the whole-slide images is presented. This study provides a concept of immune subtypes to understand the immune microenvironment of GC and make it easy-use in clinical implications, which may have benefit for personalizedimmunotherapy and prognosis evaluation.

Immune microenvironment has been confirmed to be associated with prognosis in gastric cancer. However, traditional methods simply describe the relationship between the cell composition of the immune microenvironment and prognosis according to the known outcome. Our method is ‘unsupervised’, which can better represent the complex and obscure information within the immune microenvironment. Significant survival differences are observed among the immune subtypes in this study, which can be a supplement to traditional TNM staging system. Specifically, an immune-hot subtype 3 presents with better prognosis, and by contrast, the immune-cold subtype 1 to 2 demonstrated a poor prognosis. Furthermore, the proportion EVB and MSI of the TCGA molecular subtyping in the IS3 are significantly higher than that in IS1-2, which is consistent with the previous report (48). EVB and MSI subtyping always present a more active immune response.

Appropriate classification for GC is essential to individual treatment. Several subtyping systems have been proposed in the past few decades, including the World Health Organization (WHO) classification, the Lauren’s classification, intrinsic Subtypes, Lei subtypes, The Cancer Genome Atlas (TCGA) subtypes, Asian Cancer Research Group (ACRG) subtypes, and some other additional classifications (49). Some are based on morphology or pathology, and some are based on the molecular and genetic features. However, classification based on immune data for GC is not well elaborated. Tumor-related immune information plays an important role in the development of tumors. The immune subtypes proposed in this study based on TIICs are independent of existing classifications. Interestingly, we also find that the proportion of different Lauren’s classification (intestinal and diffuse) and TCGA classification (EVB, MSI, CIN, GS) is different among three immune subtypes, which suggests an interaction between them. This underlying implication is worth further study. Besides, different from the previous classification, our immune subtypes have the underlying value to guide the immunotherapy and to predict prognosis.

The relationship between various types of immune cells as immune-suppressive and immune-promoting elements and tumor has been widely explored. The high abundance of tumor-associated lymphocytes, including CD8+ T cell, CD4+ T cell, and NK cell, plays a positive impact on prognosis of gastric cancer by dissolving tumor cells directly (22–25). Also, Tfh cells promote tumor-associated lymphocytes to play an anti-tumor role in gastric cancer by producing diverse antibodies and cytokines (23). By contrast, tumor-associated macrophages (TAMs), Tregs, B cell, and mast cells play the central role in the antitumor immune responses, such as negatively regulating T cell immunity (22–26). Besides, DC, as the key role in antigen presenting cells, had many subtypes. Some could induce the generation of CD8+ effector T cells through presenting the MHC class I molecules to T cells and some may inhibit immune response. In this study, we find that IS3 with high abundance of CD8+ T cells, NK cells, and Tfh cells have a better prognosis, and IS1-2 with high abundance of DC, Tregs, B cell, and mast cell have a poor prognosis. Interestingly, the two subtyping of CD4+ T cells show an opposite trend of aggregation in immune subtypes, which may play different immune functions. Furthermore, different research directions of immunotherapy could be suggested according to the immune subtypes. For IS3, it may be sufficient to mobilize the antitumor function of tumor-associated lymphocytes alone; whereas IS1-2, inhibition of anti-tumor immune response, and promoting the formation of tumor-associated lymphocytes are equally important in immunotherapy.

A series of classical tumor and immune-related pathways are found in the GO and GSEA analyses. For example, in our study, gastric cancer of IS3 is demonstrated with the highest enrichment of T cell receptor signaling and P53 signaling. In comparison, tumors of IS1-2 are confirmed with the highest enrichment of TGF-BETA signaling and JAK-STAT signaling. This reflects the difference in the composition of immune microenvironment and partly explains the difference in prognosis between them. More interestingly, high expression of PD1, PDL1, CTLA4, and TP53, and low expression of JAK1 are found in IS3. Currently, the most well-studied immune checkpoint inhibitors, such as ipilimumab and pembrolizumab, target at CTLA4 and PD1, then releases effector T cells from negative feedback pathway. Therefore, immune-hot IS3 tumor with high expression of CTLA4 and PD1 may respond better to current immunotherapy which should be fully considered in immunotherapy.

T cell infiltration and immune checkpoint (PD-1, PD-L1, and CTLA-4) are known as predictors to immunotherapy (22). Tumor immune microenvironment involves the interaction of multiple immune cells, which contains a more complex relationship and is closely related to immunotherapy. Relationship between T cell infiltration and immune response is not clear. The location of T cell and other immune cells (e.g. Tregs and DC) also play an important role (25, 50). Meanwhile, not all patients with positive immune checkpoints respond well to the immunotherapy (15, 16). In this study, we find that the IS1 was also infiltrated with abundant T cells, but the expression of immune checkpoint is not high, and the prognosis is poor. This may be related to its strong immunosuppression, such as high abundance of Tregs and low abundance of Tfh cells (31, 51). Meanwhile, the expression level of almost all immune-infiltrating cells, except for macrophages, is low in IS2. And M2 macrophages abundance is the highest in IS2, while M1 macrophages’ abundance is the lowest in IS2. This indicates a status of immunologic deficiency and immunosuppression (52). IS3 shows an immune-hot status with high T cell infiltration. These findings suggest that it is more valuable to study the tumor immune cell microenvironment as a whole and suggest the possibility of different immunotherapeutic strategies for different immune subtypes. For IS1, appropriate treatment targeting regulatory cells (e.g., Tregs and DC) is also important (31, 51). For IS2, in addition to enhancing immune activity, it can also be considered to promote M1 polarization of macrophages and inhibit M2 polarization to promote the immune response (52). For IS3, enhancing the function of existing T cells may be enough.

The whole transcriptome sequencing data are difficult to obtain due to its high cost. Besides, flow cytometry to detect all immune cells in the immune microenvironment is difficult and requires complex protocol and high quality of GC tissue. Thus, we hope to get information about the immune subtypes in a more convenient way. Therefore, considering the extensive and easy application of HE pathological sections in clinical practice, a deep learning model based on ResNet-18 is developed and validated for our immune subtypes based on the whole-slide image. We put forward such a conceptual framework that the immune subtype could be predicted based on the whole-slide pathological image. With limited cases, we find that deep learning can predict the immune subtypes well. In the future, if enough cases and a perfect deep learning model are available, the immune subtypes can be easily used in clinical practice.

There are several limitations to this study. First, our analysis is only focused on tumor-infiltrating immune cells, while other components in tumor microenvironment might also play important role. Second, immune infiltration cells were generated from gene expression profiles, which means the location information of immune cells could not be further analyzed. Third, the possibility of selection bias in this retrospective study could not be excluded. Fourth, gastric cancer is a highly heterogenous cancer. Three subtypes to predict the response to immunotherapy may not be enough. In the future, we will focus on the discovery of new immune subtypes for GC. Fifth, the exactly parameters used by deep learning to distinguish subtypes cannot be acquired. Finally, a small sample size should not be ignored.

In conclusion, we confirm three reproducible immune subtypes of gastric cancer. Each of the three immune subtypes possess distinct compositions of tumor immune-infiltrating cells, molecular features, and clinical characteristics. We then develop and validate a deep learning model based on pathological images to predict the immune subtypes. Our study puts forward a conceptual framework of immune subtypes to understand the immune microenvironment of gastric cancer better, which may provide references for the future design of immune-related studies and immunotherapy selection.

## Data Availability Statement

The original contributions presented in the study are included in the article/**Supplementary Material**. Further inquiries can be directed to the corresponding authors.

## Ethics Statement

This study was deemed exempt from institutional review board approval by Tongren Hospital, Shanghai Jiao Tong University, School of Medicine (Shanghai, China).

## Author Contributions

All authors listed had made a substantial contribution to the work. ZQX and SH put forward the conception and designed the study. CYL, RYC, WL and JJD collected and collated the data and do the language editing. YC, ZPS and WLC analyzed data and wrote the manuscript together. XZF, JZ and XYS made contribution to proofread the article. Finally, All authors contributed to the article and approved the submitted version.

## Funding

This work was supported by grants from: Health Commission of Changning District, Shanghai (YXMZK009) and Tongren Hospital, Shanghai Jiao Tong University, School of Medicine (TR2020xk28).

## Conflict of Interest

The authors declare that the research was conducted in the absence of any commercial or financial relationships that could be construed as a potential conflict of interest.
