# Supplementary material for: The Immune Subtypes and Landscape of Gastric Cancer and to Predict Based on the Whole-Slide Images Using Deep Learning
Source: Front Immunol. 2021 Jun 28;12:685992. doi: 10.3389/fimmu.2021.685992 (PMC8273735; doi:10.3389/fimmu.2021.685992)
Supplement: Supplementary file 1 [file DataSheet_1.docx]

Supplementary Material

The immune subtypes and landscape of gastric cancer and to predict based on the whole-slide images using deep learning

1. **Supplementary Methods**
2. **Supplementary Results**
3. **Supplementary Tables and Figures**
4. **Supplementary Reference**

Contents

1. **Supplementary Methods**

1. Study design

2. Data preprocessing

3. Definition of TCGA molecular subtyping

1. **Supplementary Results**

1. Comparison of TIICs between any two immune subtypes

1.1 IS1 vs IS2

1.2 IS1 vs IS3

1.3 IS2 vs IS3

2. Deep learning model for IS1-2 vs IS3

1. **Supplementary Tables and Figures**

Table S1. Clinicopathological characteristics of patients with gastric cancer in GEO.

Table S2. Univariable and multivariable analyses for overall survival in patients with gastric cancer in GEO.

Table S3. The expression level of immune and tumor markers in patients from TCGA and GEO.

Table S4. [Differential](javascript:;) [expression](javascript:;) genes both in TCGA and GEO based on IS1 vs IS2.

Table S5. [Differential](javascript:;) [expression](javascript:;) genes both in TCGA and GEO based on IS1 vs IS3.

Table S6. [Differential](javascript:;) [expression](javascript:;) genes both in TCGA and GEO based on IS2 vs IS3.

Figure S1. Workflow of this study.

Figure S2. The landscape of 22 tumor-infiltrating immune cells in TCGA and GEO

Figure S3. The proportions of 22 TIICs in each sample quantified by CIBERSORT.

Figure S4. The validation cohort shows heterogeneity of immune infiltration among immune subtypes.

Figure S5. Highest abundance of M0 and M2 was observed in IS2 (a, c, d, f), while lowest abundance of M1 was observed in IS2.

Figure S6. Differences in clinical characteristics among immune subtypes, including age and sex.

Figure S7. Heat map about the differentially expressed genes (DEGs) in TCGA and GEO.

Figure S8. Identification of the immune subtypes in Pan-cancer cohorts and its prognostic value.

Figure S9. The expression level of immune and tumor markers in patients from TCGA and GEO.

Figure S10. The prediction accuracy of deep learning model based on ResNet-18 for immune subtypes.

1. **Supplementary Reference**

**I Supplementary Methods**

**1. Study design**

The overall design of our study to identify and validate immune subtypes in gastric cacner and describe the deep learning model is shown in supplementary Fig. S1. Firstly, through the CIBERSORT algorithm[1], we obtain the abundance of 22 Tumor-infiltrating immune cells from the discovery cohort in TCGA-STAD. Based on the above results, we used unsupervised consensus clustering to identify immune subtypes of GC[2]. The repeatability of identified immune subtypes were then validated in an independent cohort of GSE84437. We further evaluated the clinical, molecular and cellular characteristics associated with the immune subtypes. Besides, we illustrate the feasibility of our immune subtypes based on five independent pan-cancer cohorts, including [breast](javascript:;) [cancer](javascript:;), [esophagus](javascript:;) [cancer](javascript:;), [colorectal](javascript:;) [cancer](javascript:;), [liver](javascript:;) [cancer](javascript:;) and [pancreatic](javascript:;) [cancer](javascript:;). Finally, a convolutional neural network with deep residual learning (ResNet-18) model to detect the immune subtype by transfer learning using patches segmented from the whole slide images (WSIs) was developed and validated[3].

**2. Data preprocessing**

For the discovery cohort, we selected suitable gastric cancer patients through the following inclusion and exclusion criteria in TCGA. Exclusion criteria: ⑴samples with death reason of other malignancy and non-malignant disease; ⑵sample type is not “Primary Tumor”; ⑶samples with pathological type marked as “neoplasms, nos”, “epithelial neoplasms, nos” or “mature b-cell lymphomas”; ⑷ samples with incomplete overall survival information and overall survival of follow-up less than 30 days. ⑸The pathological stage is unknown. ⑹ Lack of information of immune score or stromal score. Inclusion criteria: ⑴ sample type is “Primary Tumor”; ⑵ samples marked as “TCGA-STAD”; ⑶ samples with complete gene expression profile. For the independent validation cohort of GSE84437, gene expression data of the Illumina platform and clinical information were retrieved from the GEO database. For the pan-cancer validation cohorts, gene expression profile and survival data were download from UCSC Xena. Then, gene expression data in discovery and validation cohort were normalized using “limma” R package before subsequent processing.

**3. Definition of** **TCGA molecular subtyping**

 Multiple genomic and proteomic data, including somatic mutations, mRNA expression, miRNA expression, promoter methylation, somatic copy-number alteration and protein expression data of the TCGA gastric cancer cohort were processed by the TCGA working team. According to the TCGA classification scheme employed a decision tree whereby gastric tumors were divided into four subtypes[4]. Briefly, tumors were first categorized by the presence of EBV features (EBV subtype), then by the presence of high MSI (MSI subtype). The remaining tumors were further grouped by the number of somatic copy-number alterations: genomically stable (GS subtype) or chromosomal instability (CIN subtype). Afterwards, we identify 376 cases with confirmed information of TCGA molecular subtyping from [cBioPortal for Cancer Genomics](http://www.cbioportal.org/) database (http://www.cbioportal.org/), including 30 EVB, 73 MSI, 50 GS and 223 CIN. Finally, both molecular subtyping and immune subtypes data were available in 192 samples, including 22 EVB, 38 MSI, 37 GS and 95 CIN, in this study.

**II Supplementary Results**

**1. Comparison of TIICs between any two immune subtypes**

**1.1 IS1 vs IS2**

Immune subtypes 1 and immune subtypes 2 represented distinct immune cells expression patterns in the discovery cohort, which was found to be highly consistent with the validation cohort, surprisingly. Compared to IS2, a higher CD8+ T cells, CD4+ T memory resting cells, B cells, DCs, Regulatory T cell abundance and M1 macrophages was confirmed in IS1 **(Figure 2a, Figure 2b, Figure 2d, Figure 2e and Figure 2i, Figure S4a, Figure S4b, Figure S4d, Figure S4e, Figure S4i, Figure S5b and Figure S5e)**. In comparison, a lower follicular helper T cells, M0 macrophages and M2 macrophages abundance was found in IS1 (**Figure 2f and Figure 2h, Figure S4f, Figure S4h and Figure S5a, Figure S5d, Figure S5c and Figure S5f**). However, the expression of CD4+ T memory activated cells and NK cells in IS1 was higher than that in IS2 **(Figure 2c and Figure 2j)**, and the expression of Mast cells in IS1 was lower than that in IS2 in the discovery cohort (**Figure 2g)**, which showed the opposite trend in the validation cohort (**Figure S4c, Figure S4g and Figure S4j**). Lastly, we identified IS1 exhibited higher immune score and higher stromal score compared with IS2 (**Figure 2k and Figure 2l**).

**1.2 IS1 vs IS3**

Immune subtypes 1 and immune subtypes 3 represented distinct immune cells expression patterns in the discovery cohort, which was found to be highly consistent with the validation cohort, surprisingly. Compared to IS1, a higher CD8+ T cells, CD4+ T memory activated cells, follicular helper T cells, NK cells and M1 macrophages abundance was confirmed in IS3 **(Figure 2a, Figure 2c, Figure 2h and Figure 2j, Figure S4a, Figure S4c, Figure S4h, Figure S4j, Figure S5b and Figure S5e)**. In comparison, a lower CD4+ T memory resting cells, B cells, DCs, Mast cells, Regulatory T cell and M2 macrophages abundance was found in IS3 (**Figure 2b, Figure 2d, Figure 2e, Figure 2g and Figure 2i, Figure S4b, Figure S4d, Figure S4e, Figure S4g, Figure S4i, Figure S5c and Figure S5f**). However, the expression of macrophages in IS3 was higher than that in IS1 **(Figure 2f)**, and the expression of Plasma cells and M0 macrophages in IS3 was lower than that in IS1 in the discovery cohort (**Figure S4l and Figure S5a),** which showed the opposite trend in the validation cohort (**Figure S4f and Figure S4k and Figure S5d**). Lastly, we identified IS3 exhibited higher immune score and lower stromal score compared with IS1 (**Figure 2k and Figure 2l**).

**1.3 IS2 vs IS3**

Immune subtypes 2 and immune subtypes 3 represented distinct immune cells expression patterns in the discovery cohort, which was found to be highly consistent with the validation cohort, surprisingly. Compared to IS2, a higher CD8+ T cells, CD4+ T memory activated cells, DCs, follicular helper T cells, NK cells, Plasma cells abundance and M1 macrophages was confirmed in IS3 **(Figure 2a, Figure 2c, Figure 2e, Figure 2h, Figure 2j and Figure S4l, Figure S4a, Figure S4c, Figure S4e, Figure S4h, Figure S4j, Figure S4k, Figure S5b and Figure S5e)**. In comparison, a lower CD4+ T memory resting cells, B cells, macrophages and Mast cells, M0 macrophages and M2 macrophages abundance was found in IS3 (**Figure 2b, Figure 2d, Figure 2f and Figure 2g, Figure S4b, Figure S4d, Figure S4f, Figure S4g and Figure S5a, Figure S5d, Figure S5c and Figure S5f**). However, the expression of Regulatory T cell in IS3 was higher than that in IS2 **(Figure 2i),** which showed the opposite trend in the validation cohort (**Figure S4i**). Lastly, we identified IS3 exhibited higher immune score and lower stromal score compared with IS2 (**Figure 2k and Figure 2l**).

**1. Deep learning model for IS1-2 vs IS3**

After removing low quality pathological images, 164 samples with WISs were divided into training (84 cases), validation (51 cases) and test cohort (34 cases), and then tumor ROI was separated into 512×512 patches. Finally, the training cohort contained 16385 normalized tiles marked as IS1-2 and 6323 normalized tiles marked as IS3. The validation cohort contained 14073 normalized tiles marked as IS1-2, 5114 normalized tiles marked as IS3. And the test cohort contained 7134 normalized tiles marked as IS1-2 and 2508 normalized tiles marked as IS3. Next, we developed a ResNet-18 deep learning model to predict the immune subtypes based on training and validation, and measured the performance in the test cohort. The model first predicted the probability of immune subtypes for each patch. We found that the accuracy of IS prediction for each patch in the training, validation and test cohort was 83.36%, 72.14% and 68.45% respectively. Then GC cases would be designated as one of the two subtypes (IS1-2 or IS3) according to the accumulated number of patches in tumor ROI. We observed that the accuracy of IS prediction ResNet-18 model for GC cases was about 92.85%, 90.19%, 88.24% in the training, validation and test cohorts, separately **(Figure S10d and Figure S10e and Figure S10f)**.

**III. Supplementary Tables and Figures**

| Table S1. Clinicopathological characteristics of patients with gastric cancer in GEO | | | | | | |
| --- | --- | --- | --- | --- | --- | --- |
| **Variables** | GEO | | | | | |
|  | IS1 (n=153) | | IS2 (m=59) | | IS3 (n=87) | |
|  | N | % | N | % | N | % |
| **Age (median, IQR, Y)** | 58 (48-66) | | 64 (55-69) | | 64 (55-71) | |
| **Gender** |  |  |  |  |  |  |
| Male | 105 | 68.6 | 39 | 66.1 | 57 | 65.5 |
| Female | 48 | 31.4 | 20 | 33.9 | 30 | 34.5 |

| Table S2. Univariable and multivariable analyses for overall survival in patients with gastric cancer in GEO | | | | |
| --- | --- | --- | --- | --- |
| **Variables** | Univariable analysis (N=194) | | Multivariable analysis (N=194) | |
|  | OR (95%CI) | **P** | OR (95%CI) | **P** |
| **Age** (years) | 1.018 (1.004-1.033) | 0.012 | 1.025 (1.011-1.040) | 0.001 |
| **Gender** (female vs. male) | 1.244 (0.861-1.798) | 0.245 | NA | NA |
| **T stage (**T1 vs T2 vs T3 vs T4) | 1.645 (1.246-2.173) | <0.001 | 1.609 (1.205-2.151) | 0.001 |
| **N stage (**N1 vs N2 vs N3) | 1.649 (1.357-2.005) | <0.001 | 1.609 (1.205-2.151) | <0.001 |
| **Immune subtype** |  |  |  |  |
| IS1 | 1 | NA | 1 | NA |
| IS2 | 0.925 (0.604-1.418) | 0.721 | 0.747 (0.435-1.282) | 0.289 |
| IS3 | 0.624 (0.409-0.951) | 0.028 | 0.491 (0.282-0.853) | 0.012 |

| - Table S3. The expression level of immune and tumor markers in patients from TCGA and GEO | | | | | | | | |
| --- | --- | --- | --- | --- | --- | --- | --- | --- |
| **Variables** | TCGA | | | | GEO | | | |
|  | IS1 (n=99) | IS2 (n=43) | IS3 (n=52) | P value | IS1 (n=153) | IS2 (n=59) | IS3 (n=87) | P value |
|  | median (IQR) | median (IQR) | median (IQR) |  | median (IQR) | median (IQR) | median (IQR) |  |
| **PD1** | 218 (110-437) | 124 (85-246) | 348 (219-521) | <0.001 | 82 (68-98) | 80 (64-101) | 122 (88-167) | <0.001 |
| **PD-L1** | 345 (215-502) | 378 (188-658) | 627 (264-1605) | 0.001 | 63 (58-71) | 66 (61-75) | 83 (66-110) | <0.001 |
| **CTLA4** | 213 (108-369) | 202 (76-310) | 238 (181-449) | 0.044 | 138 (113-179) | 163 (121-201) | 216 (170-271) | <0.001 |
| **TP53** | 4987 (2786-7529) | 5303 (3379-8320) | 6192 (3924-8534) | 0.127 | 145 (108-182) | 140 (95-208) | 194 (108-261) | 0.014 |
| **JAK1** | 13170 (9555-18272) | 12026 (5733-16956) | 11537 (8903-18839) | 0.548 | 706 (554-949) | 625 (460-817) | 613 (424-917) | 0.042 |

| - Table S4. [Differential](javascript:;) [expression](javascript:;) genes both in TCGA and GEO based on IS1 vs IS2. | | | | | | | | |
| --- | --- | --- | --- | --- | --- | --- | --- | --- |
| IGF2 | CCNE1 | MMP9 | COL7A1 | LY6G6D | NKD1 | NFE2 | APOA2 | CES1 |
| NOTUM | TUBB2B | VTN | XAGE1B | MMP12 | APCDD1 | SCG3 | PAGE2B | TTYH1 |
| MMP3 | PAGE2 | MAGEA10 | GNG4 | TRIB3 | MYBPHL | GPR37 | GLDC | VGF |
| SALL4 | TF | PNMA3 | MAGEA4 | APOA1 | KRT23 | CSAG1 | FGG | VPREB3 |
| FCRLA | CCL19 | MAL | BANK1 | CCR7 | CXCL13 | CD79A | GHRL | FCER1A |
| ADH1A | ADH1B | PTGDS | CHIA | GREM2 | FHL1 | PI16 | ABI3BP | PGA5 |
| CASQ2 | MGP | JAM2 | FAM107A | VIP | OGN | SYNPO2 | CRYAB | PGA4 |
| ATP4A | KLRB1 | ABCA8 | FLNC | THBS4 | IGF1 | RCAN2 | OMD | GAST |
| SRPX | ODAM | MYLK | C2orf40 | PODN | LMOD1 | SFRP1 | CFD | KRT13 |
| COL14A1 | ATP4B | PGM5 | APOA4 | HDC | CNN1 | HSPB6 | MMRN1 | KCNE2 |
| LIPF | MYL9 | KCNMB1 | C6orf58 | HSPB7 | CILP | MAMDC2 | PDK4 | CPA2 |
| RBPMS2 | SCRG1 | LTF | HTR2B | DES | RGMA | SMYD1 | TACR2 | MRGPRD |
| MRGPRF | CTSG | GIF | C7 | GCG | BCHE | SST | ADH1C |  |

| - Table S5. [Differential](javascript:;) [expression](javascript:;) genes both in TCGA and GEO based on IS1 vs IS3. | | | | | | | | |
| --- | --- | --- | --- | --- | --- | --- | --- | --- |
| GZMH | CXCL10 | WARS | CXCL11 | GBP5 | CCL5 | NKG7 | LAG3 | GZMB |
| IFNG | GBP1 | CD8A | ZNF683 | PRF1 | GBP4 | CX3CL1 | GZMA | RARRES3 |
| CXCL9 | BATF2 | HAPLN3 | UBD | IL32 | GNLY | AIM2 | PRODH | CCL8 |
| C4BPA | MUC16 | GC | SLCO1B3 | CHIA | PCSK1 | CHGB | GHRL | MAL |
| PCP4 | SLC26A3 | BCHE | PGA4 | SPON1 | C14orf132 | CEL | FCER1A | ATP4A |
| MATN2 | MYOM1 | INSM1 | ADH4 | CHGA | CPA3 | FLNC | LIPF | GREM2 |
| CA4 | FHL1 | MT1H | HSPB8 | ATP4B | MSRB3 | CAPN9 | MFAP4 | COL14A1 |
| ABCA8 | PGA5 | PDLIM3 | PPP1R3C | AQP2 | CTSG | MRGPRF | IGF1 | SYNPO2 |
| MAMDC2 | PGM5 | PGC | MYLK | SORBS1 | HMGCS2 | SMOC2 | TM4SF20 | SPARCL1 |
| GIF | TMEM100 | KCNE2 | SMTN | GKN1 | PDK4 | SCGB2A1 | REEP1 | TMOD1 |
| DEFA5 | LMOD1 | SFRP1 | CPA2 | CNN1 | RPRM | OGN | C7 | DEFA6 |
| NKX6-2 | HSPB6 | FAM3B | SMYD1 | SHISA3 | RBPMS2 | RGMA | MYL9 | CHRNA3 |
| MT1G | SI | HSPB7 | PNCK | GKN2 | KCNMB1 | TFF2 | DES | SCRG1 |
| TCEAL2 | ANPEP | ADH1B | ALDOB | LTF | PSCA | PCDH20 |  |  |

| - Table S6. [Differential](javascript:;) [expression](javascript:;) genes both in TCGA and GEO based on IS2 vs IS3. | | | | | | | | |
| --- | --- | --- | --- | --- | --- | --- | --- | --- |
| CD8A | GZMH | NKG7 | CCL5 | ZNF683 | CXCL11 | CD2 | EOMES | GZMK |
| CD96 | CXCL10 | APOBEC3G | CD3D | CXCR6 | TRIM22 | UBD | RARRES3 | TBC1D10C |
| CD6 | CX3CL1 | ITK | GZMB | HLA-DPB1 | IFNG | HLA-DMA | IL2RB | GBP5 |
| CXCL9 | CD74 | IRF1 | HLA-DOA | BATF2 | WARS | GZMA | CD247 | MYBPC2 |
| PRF1 | IL32 | SAMD9L | CXCL13 | SP140 | HLA-DOB | GBP1 | IFI44L | LAG3 |
| HLA-DQA1 | HLA-DQB2 | CD38 | GNLY | JAK2 | CASP1 | ART3 | KIR2DL3 | IKZF1 |
| CCL19 | AIM2 | SPIB | ADH1A | GBP4 | OASL | NTS | ANXA8 | KRT13 |
| MIA | MYBPC1 | PCSK1 | CHGB | NOTUM | MYT1 | APCDD1 | INHBB | CCNE1 |
| SCG3 | NKD1 | SLC29A4 | TF | TTYH1 | F10 | SERPIND1 | NEU4 | DACH1 |
| BMP7 | CXCL6 | MMP9 | BEX1 | HES6 | KRT23 | MMP3 | APOA2 | CES1 |
| MLLT11 | TUBB2B | VGF | NKD2 | CA4 | GNG4 | PCP4 | PROX1 | GLDC |
| ADAMTSL2 | SNTB1 | MEP1A | QPRT | HOXD1 | AMACR | SERPINA5 | SLC35D3 | KIF1A |
| UCHL1 | FZD9 | AGT | ITLN1 | MUC12 | MUC2 | REG1B | LY6G6D | CDH2 |
| OGDHL | FAM3B | APOA1 | TM4SF20 | IGF2 | CHST13 | DEFA5 | VANGL2 | MYBPHL |
| CEL | MAP7D2 | SERPINA1 | SI | DLX5 | ORM1 | HMGCS2 | GPR37 | SNCAIP |
| PPP1R14C |  |  |  |  |  |  |  |  |


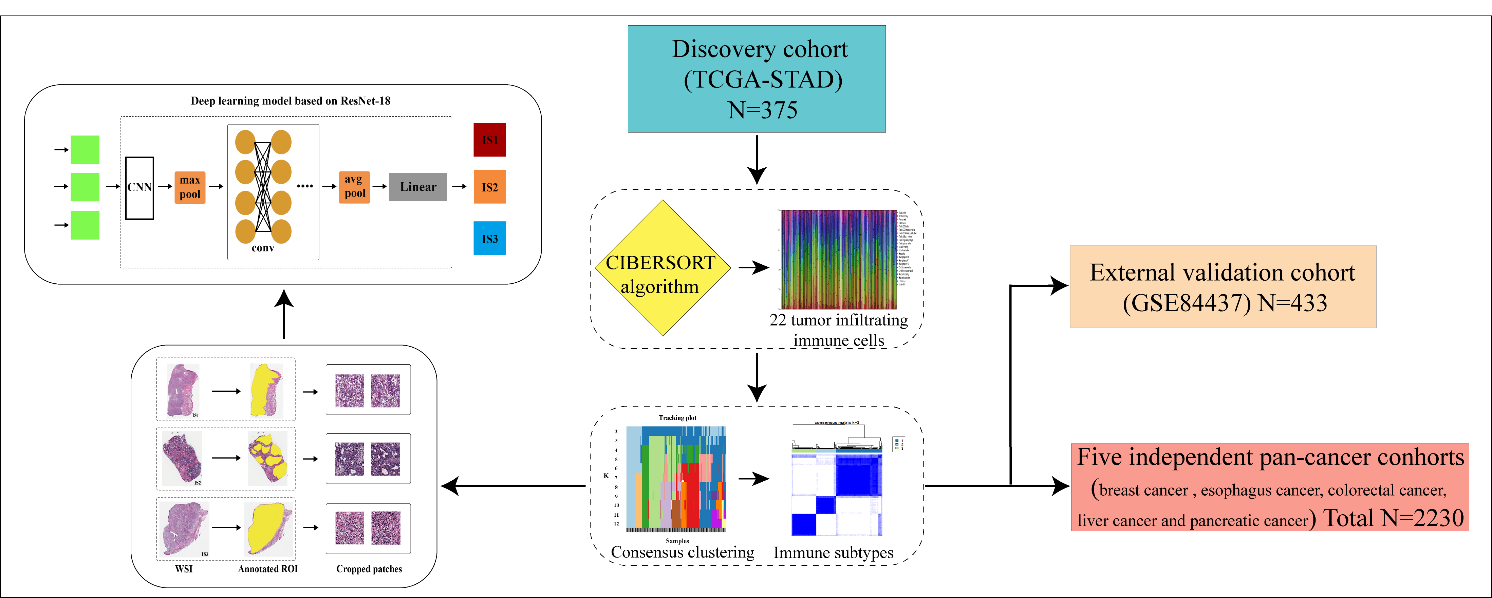


Figure S1. Workflow of this study.


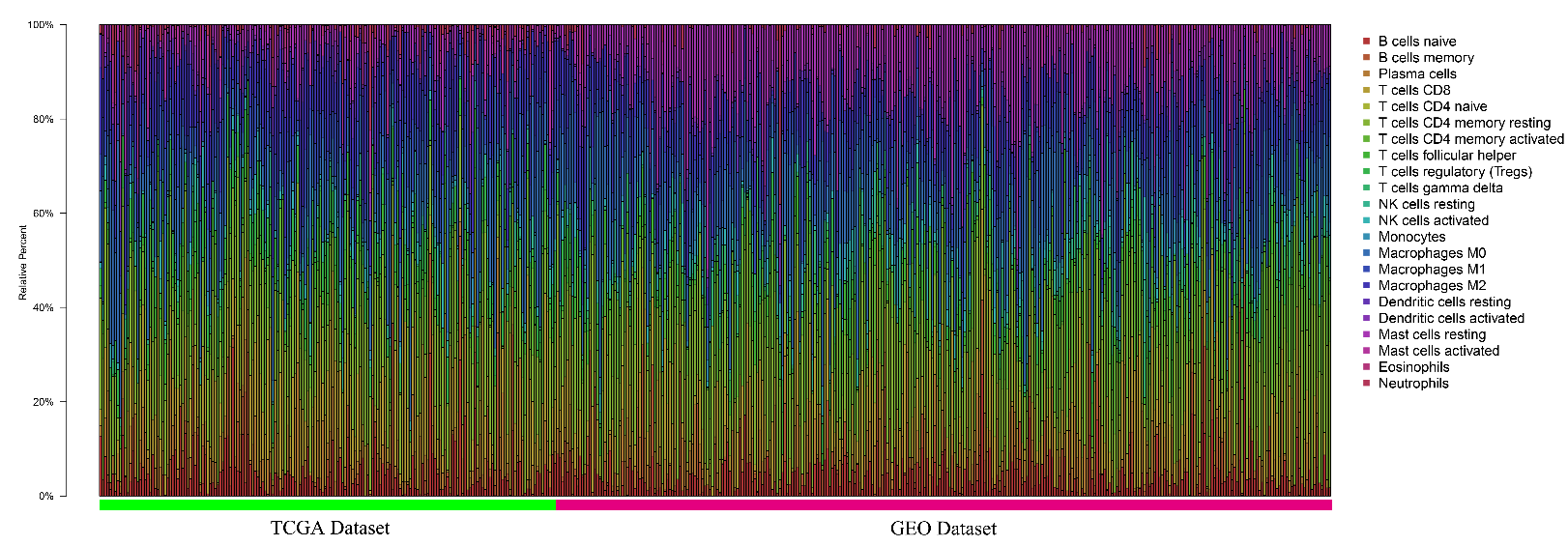


Figure S2. The landscape of 22 tumor-infiltrating immune cells in TCGA and GEO.


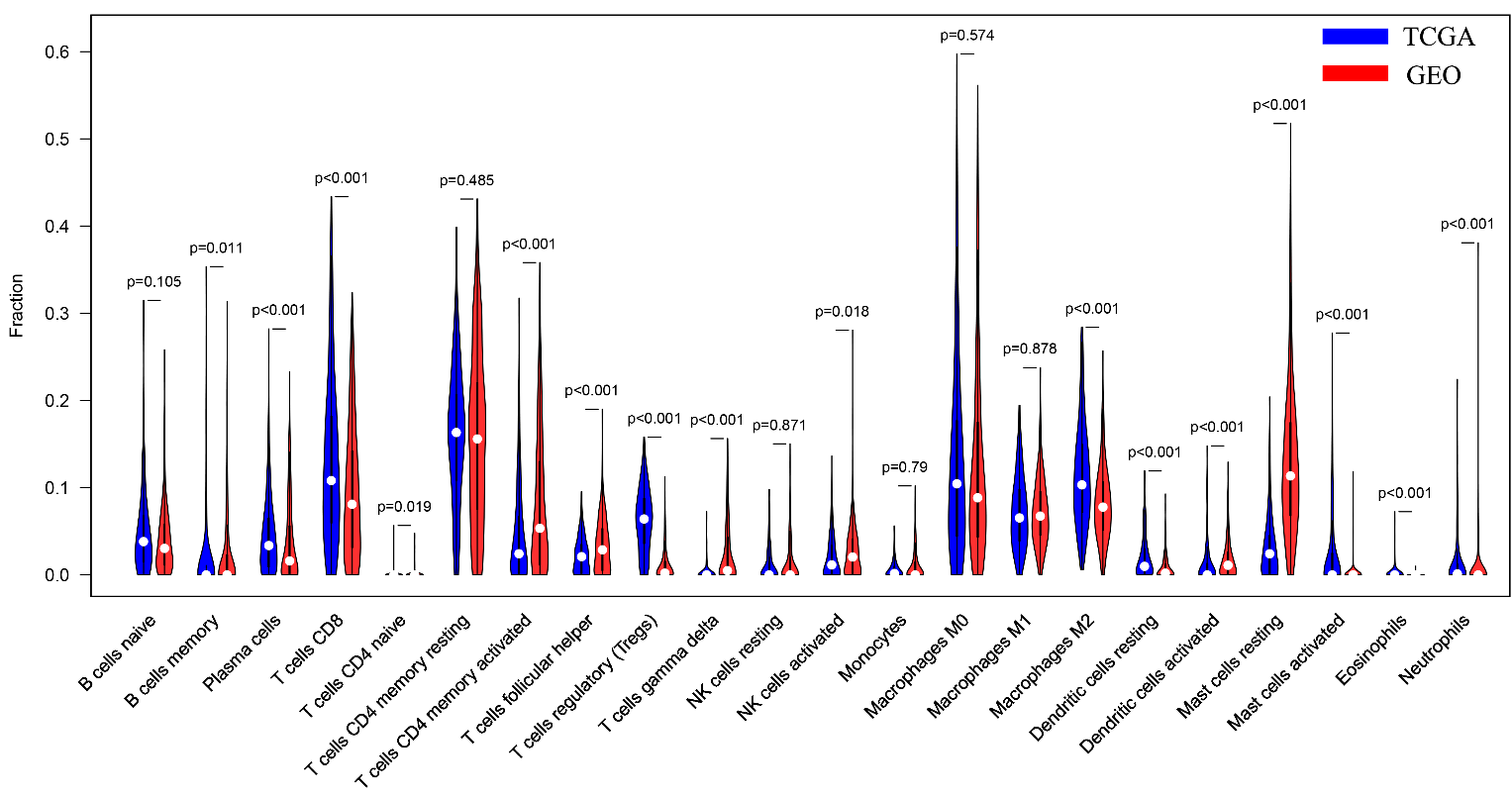


Figure S3. The proportions of 22 TIICs in each sample quantified by CIBERSORT.


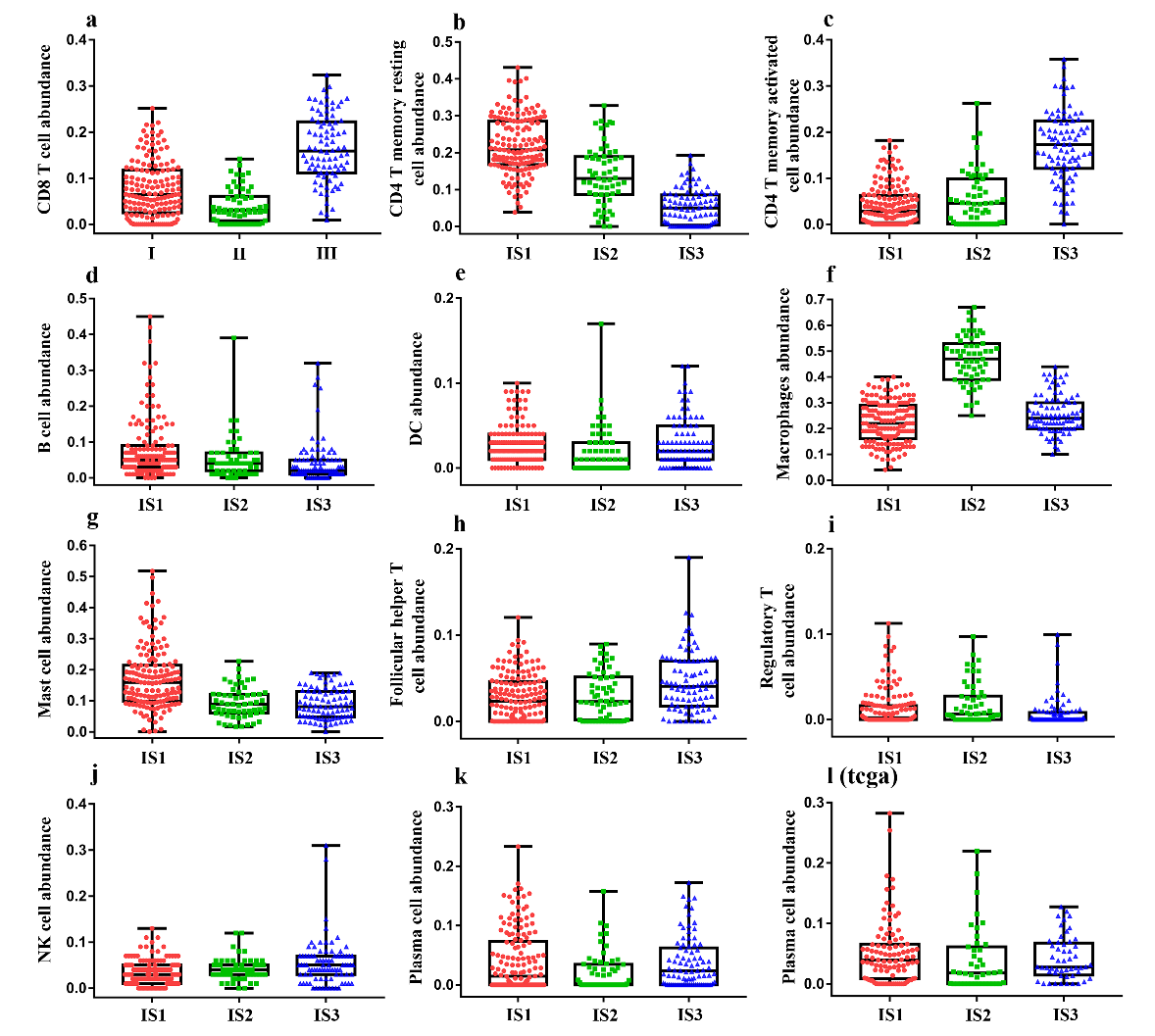


Figure S4. The validation cohort shows heterogeneity of immune infiltration among immune subtypes. Highest abundance of CD8+ T cells, CD4+ T memory activated cells, follicular helper T cells and NK cells was observed in IS3 (a, c, h, j), while lowest abundance of CD4+ T memory resting cells, B cells, macrophages cells, regulatory T cells and mast cells was observed in IS3 (b, d, f, g, i).   DCs and plasma cells abundance showed one highest and one lowest in IS1 and IS2 (e, k, l). The plot of patient immune cells abundance shows the median, 25th and 75th percentile values (horizontal bar, bottom and top bounds of the box), and the highest and lowest values (top and bottom whiskers, respectively)


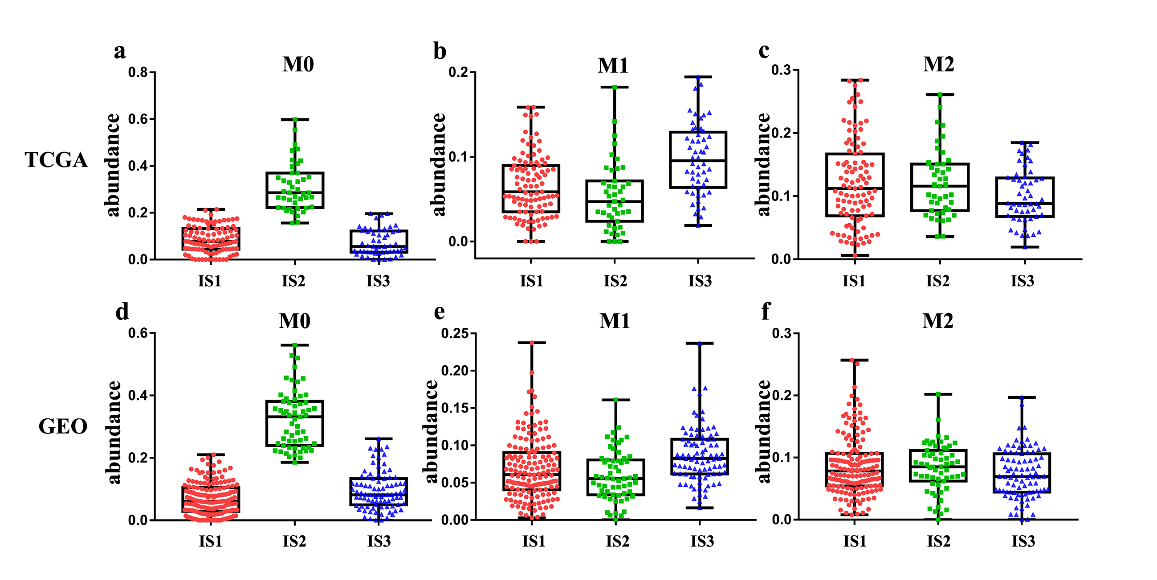


Figure S5. Highest abundance of M0 and M2 was observed in IS2 (a, c, d, f), while lowest abundance of M1 was observed in IS2 (b, e); Highest abundance of M1 was observed in IS3 (b , e), while lowest abundance of M2 was observed in IS3 (c , f).


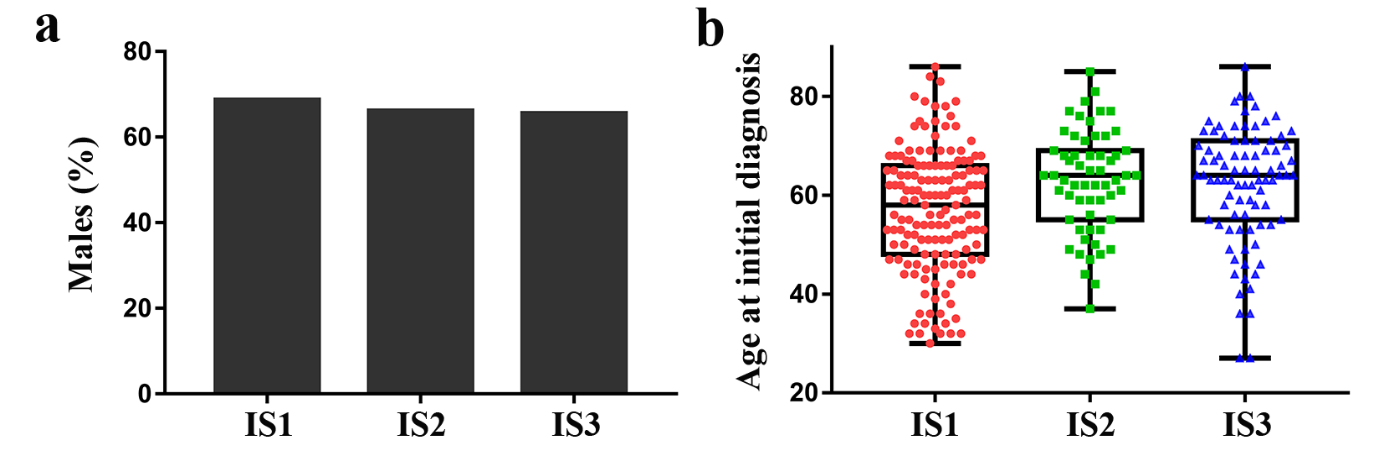


Figure S6. Differences in clinical characteristics among immune subtypes, including age and sex.


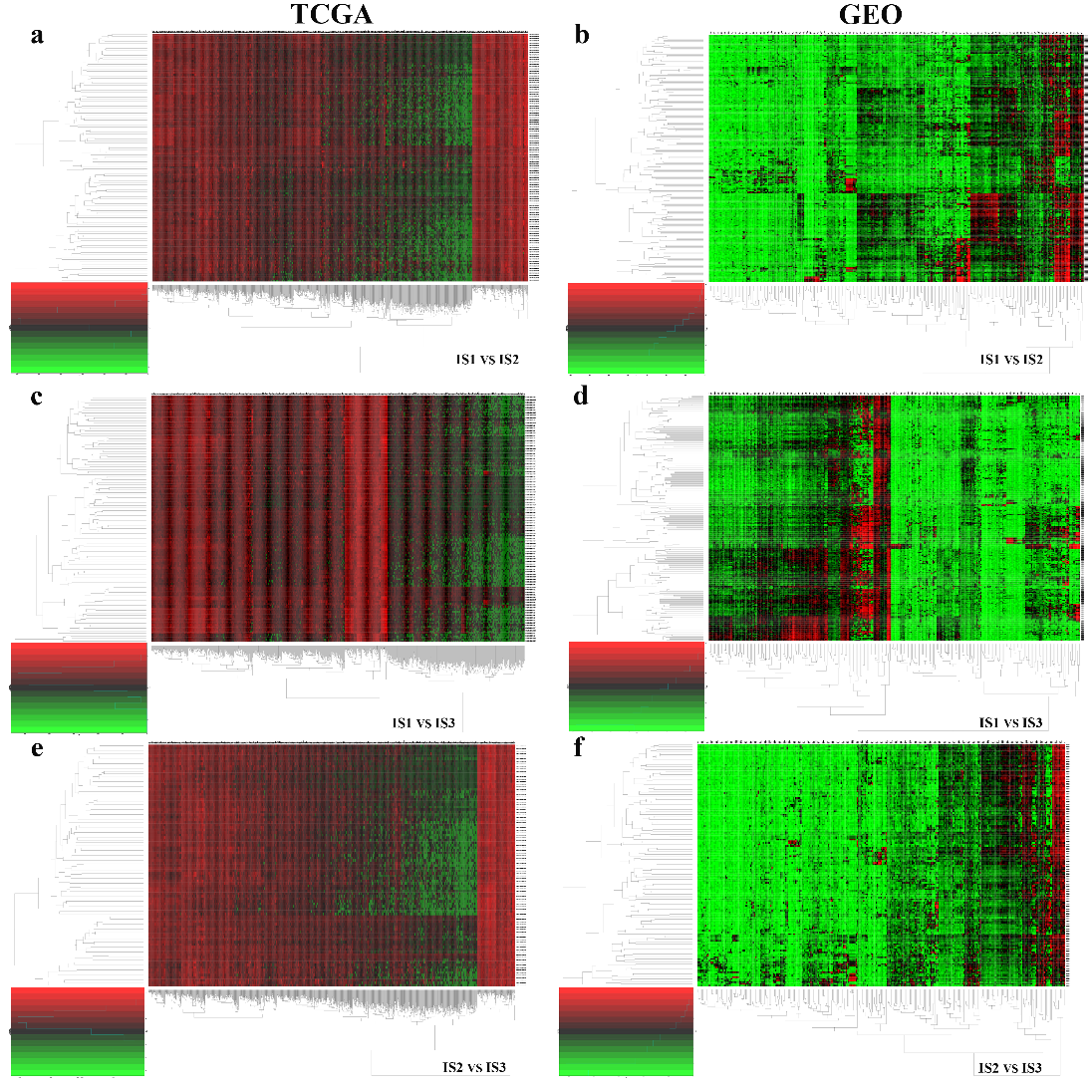


Figure S7. Heat map about the differentially expressed genes (DEGs) in TCGA (A) and GEO (B).


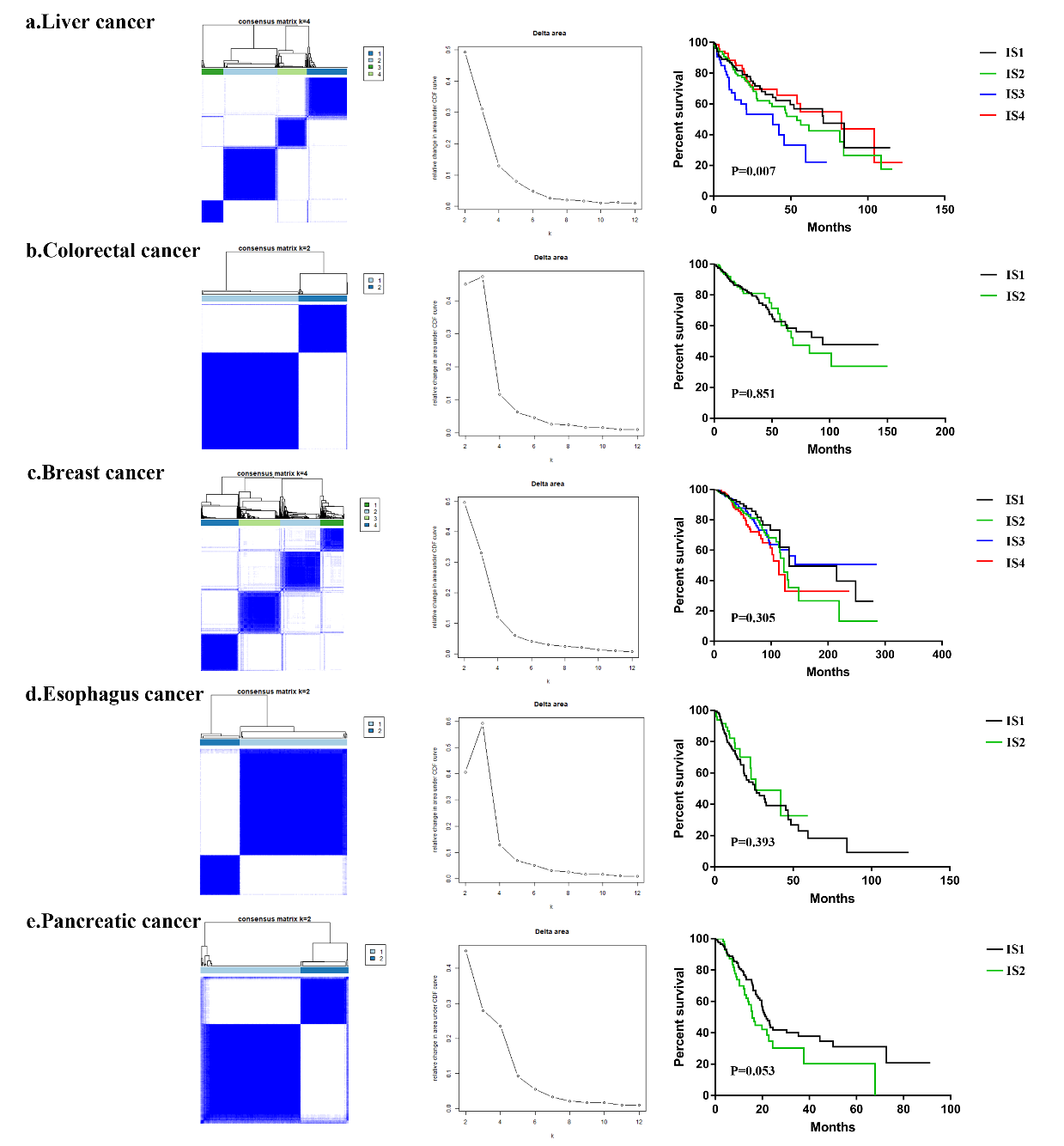


Figure S8. Identification of the immune subtypes in Pan-cancer cohorts and its prognostic value, including liver cancer, colorectal cancer, breast cancer, esophagus cancer and pancreatic cancer (a, b, c, d, e).


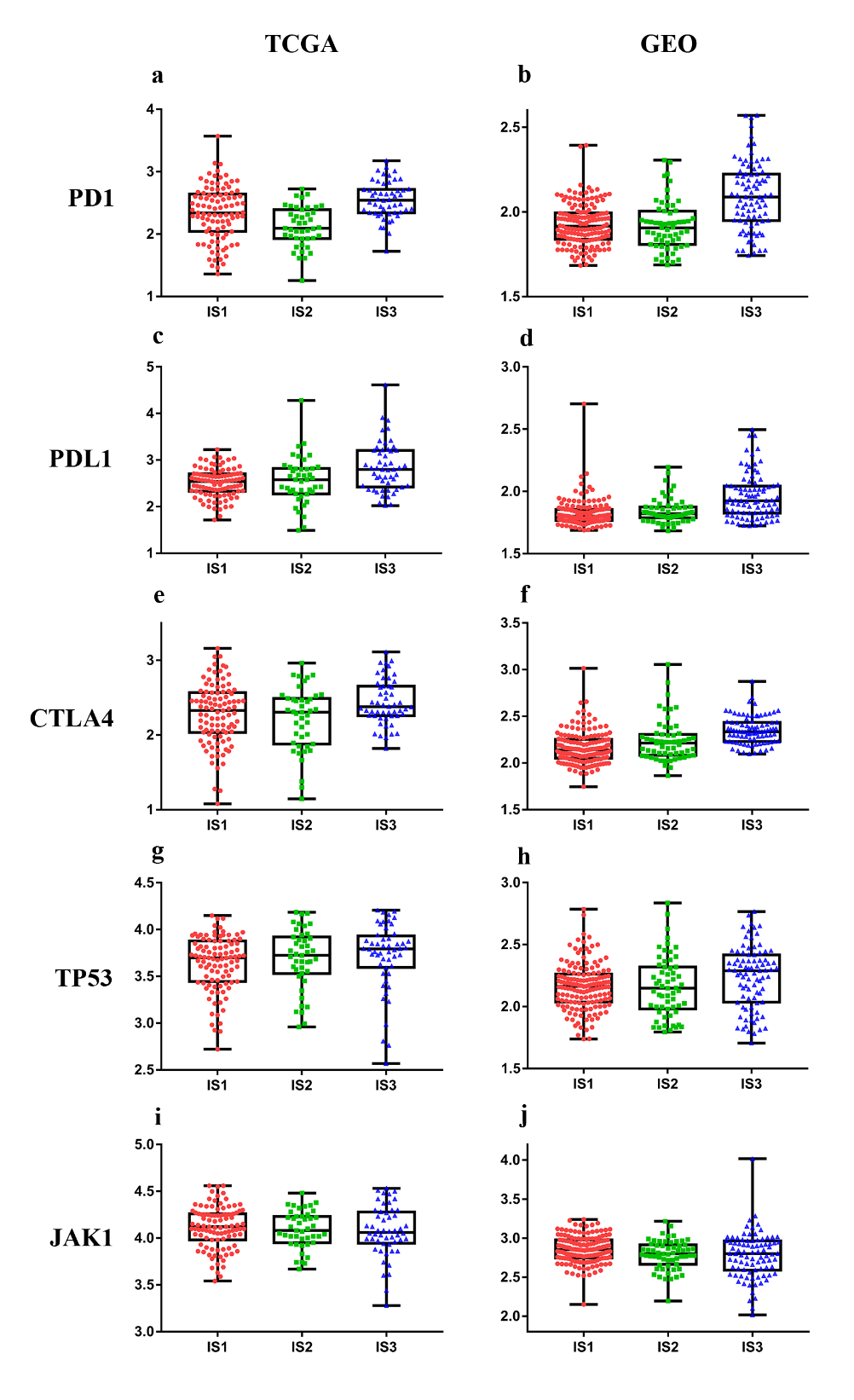


Figure S9. The expression level of immune and tumor markers in patients from TCGA and GEO, including PD1, PD-L1, CTLA4, TP53 and JAK1.


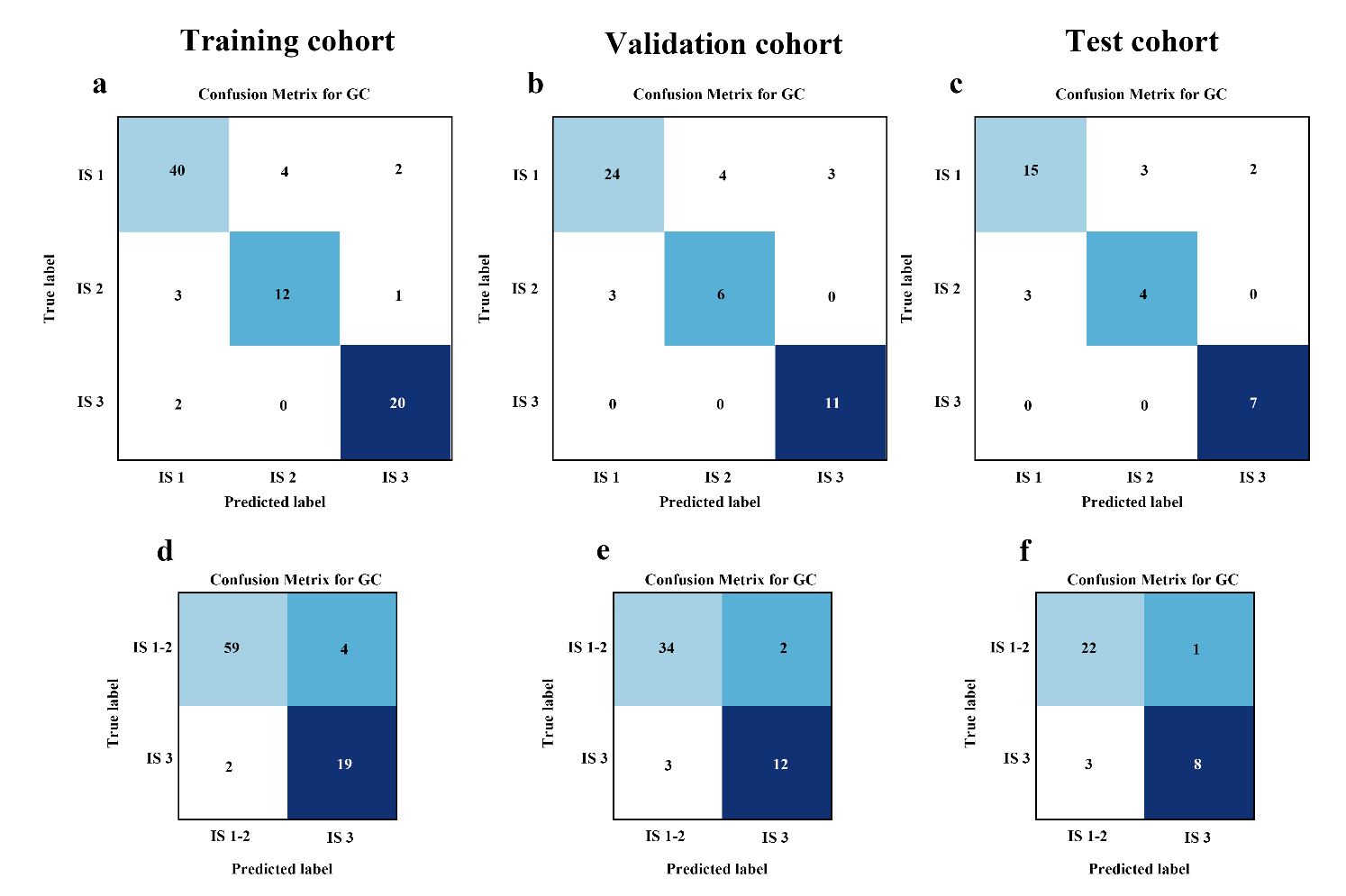


Figure S10. The prediction accuracy of deep learning model for IS1 vs IS2 vs IS3 in training (a), validation (b) and test cohort (c). And the prediction accuracy of deep learning model for IS1-2 vs vs IS3 in training (d), validation (e) and test cohort (f).

**IV Supplementary Reference**

**References:**

1. Newman, A.M., et al., *Robust enumeration of cell subsets from tissue expression profiles.* Nat Methods, 2015. **12**(5): p. 453-7.

2. Wilkerson, M.D. and D.N. Hayes, *ConsensusClusterPlus: a class discovery tool with confidence assessments and item tracking.* Bioinformatics, 2010. **26**(12): p. 1572-3.

3. Kather, J.N., et al., *Deep learning can predict microsatellite instability directly from histology in gastrointestinal cancer.* Nature medicine, 2019. **25**(7).

4. Cancer Genome Atlas Research, N., *Comprehensive molecular characterization of gastric adenocarcinoma.* Nature, 2014. **513**(7517): p. 202-9.
